# Stakeholders’ Perspectives on the Application of New Diagnostic Devices for Urinary Schistosomiasis in Oyo State, Nigeria: A Q-Methodology Approach

**DOI:** 10.9745/GHSP-D-21-00780

**Published:** 2022-08-30

**Authors:** Karlheinz Tondo Samenjo, Michel Bengtson, Adeola Onasanya, Juan Carlo Intriago Zambrano, Opeyemi Oladunni, Oladimeji Oladepo, Jo van Engelen, Jan-Carel Diehl

**Affiliations:** a Department of Sustainable Design Engineering, Faculty of Industrial Design Engineering, Delft University of Technology, Delft, Netherlands.; b Department of Parasitology, Leiden University Medical Center, Leiden, Netherlands.; c Department of Water Management, Faculty of Civil Engineering and Geosciences, Delft University of Technology, Delft, Netherlands.; d Department of Health Promotion and Education, Faculty of Public Health, College of Medicine, University of Ibadan, Ibadan, Nigeria.

## Abstract

Urinary schistosomiasis is a waterborne parasitic infection caused by *Schistosoma haematobium* that affects approximately 30 million people annually in Nigeria. Treatment and eradication of this infection require effective diagnostics. However, current diagnostic tests have critical shortcomings and consequently are of limited value to stakeholders throughout the health care system who are involved in targeting the diagnosis and subsequent control of schistosomiasis. New diagnostic devices that fit the local health care infrastructure and support the different stakeholder diagnostic strategies remain a critical need. This study focuses on understanding, by means of Q-methodology, the context of use and application of a new diagnostic device that is needed to effectively diagnose urinary schistosomiasis in Oyo State, Nigeria. Q-methodology is a technique that investigates subjectivity by exploring how stakeholders rank-order opinion statements about a phenomenon. In this study, 40 statements were administered to evaluate stakeholder perspectives on the context of use and application of potential new diagnostic devices and how these perspectives or viewpoints are shared with other stakeholders. Potential new diagnostic devices will need to be deployable to remote or distant communities, be affordable, identify and confirm infection status before treatment in patients whose diagnosis of urinary schistosomiasis is based on self-reporting, and equip health care facilities with diagnostic devices optimized for the local setting while requiring local minimal infrastructural settings. Similarly, the context of use and application of a potential new diagnostic device for urinary schistosomiasis is primarily associated with the tasks stakeholders throughout the health care system perform or procedures employed. These findings will guide the development of new diagnostic devices for schistosomiasis that match the contextual landscape and diagnostic strategies in Oyo.

## Abstract (precis)

New diagnostic devices for schistosomiasis should be designed to function best within the local endemic health care context and support stakeholders at various levels of the health care system in performing the tasks to help control and eventually eliminate schistosomiasis.

## Teaser

**Key Findings:**

- Q-methodology served as a rigorous tool to identify the needs and product-service design requirements of stakeholders throughout the health care system.
- Stakeholders at various levels of the health care system shared that new diagnostic devices for schistosomiasis should: be available at the point of need and require minimal infrastructure to increase diagnostic capacity and surveillance, particularly in rural or distant settings; be less expensive to make diagnostic testing more affordable; and be able to identify infection status before treatment to support the identification and prioritization of patients or areas in need of treatment.

**Key Implications:**

- Medical device designers and technology companies should ensure the development of new diagnostic devices for health care facilities with minimal infrastructure; these devices should be affordable and deployable, as well as able to identify infection status before treatment.
- Health care systems and community stakeholders in Nigeria should continuously participate and collaborate with the design industry in the human-centered design process of new diagnostic devices to ensure that the viewpoints of stakeholders at various levels of the health care system are explored and considered in the design process.

## INTRODUCTION

Urinary schistosomiasis is a water-borne parasitic infection caused by *Schistosoma haematobium.* This disease is prevalent in Nigeria, especially in rural areas, and affects approximately 30 million people annually.^1^^–^^7^ In specific regions, such as Oyo State, urinary schistosomiasis has an estimated prevalence of more than 50%.^2^^,^^8^^,^^9^ Many studies on *S. haematobium* are predominately school based and report a disease prevalence of 17%–21% in urban areas and 32.7% in rural areas.^10^^–^^12^

Efforts to control and eliminate schistosomiasis involve diagnosing individuals and gathering prevalence data that can be used for strategy development, program planning, and monitoring.^13^ In Oyo, schistosomiasis is addressed through 2 approaches: (1) individual case management and (2) control and elimination.^2^^,^^5^^,^^14^ In the individual case management approach, diagnosis by conventional microscopy followed by treatment is performed at a primary health care (PHC) facility.^15^^–^^17^ In the control and elimination approach, the procedure involves the surveillance of high-risk groups and areas, followed by treatment to the entire group.^18^^,^^19^

**FIGURE 1:**
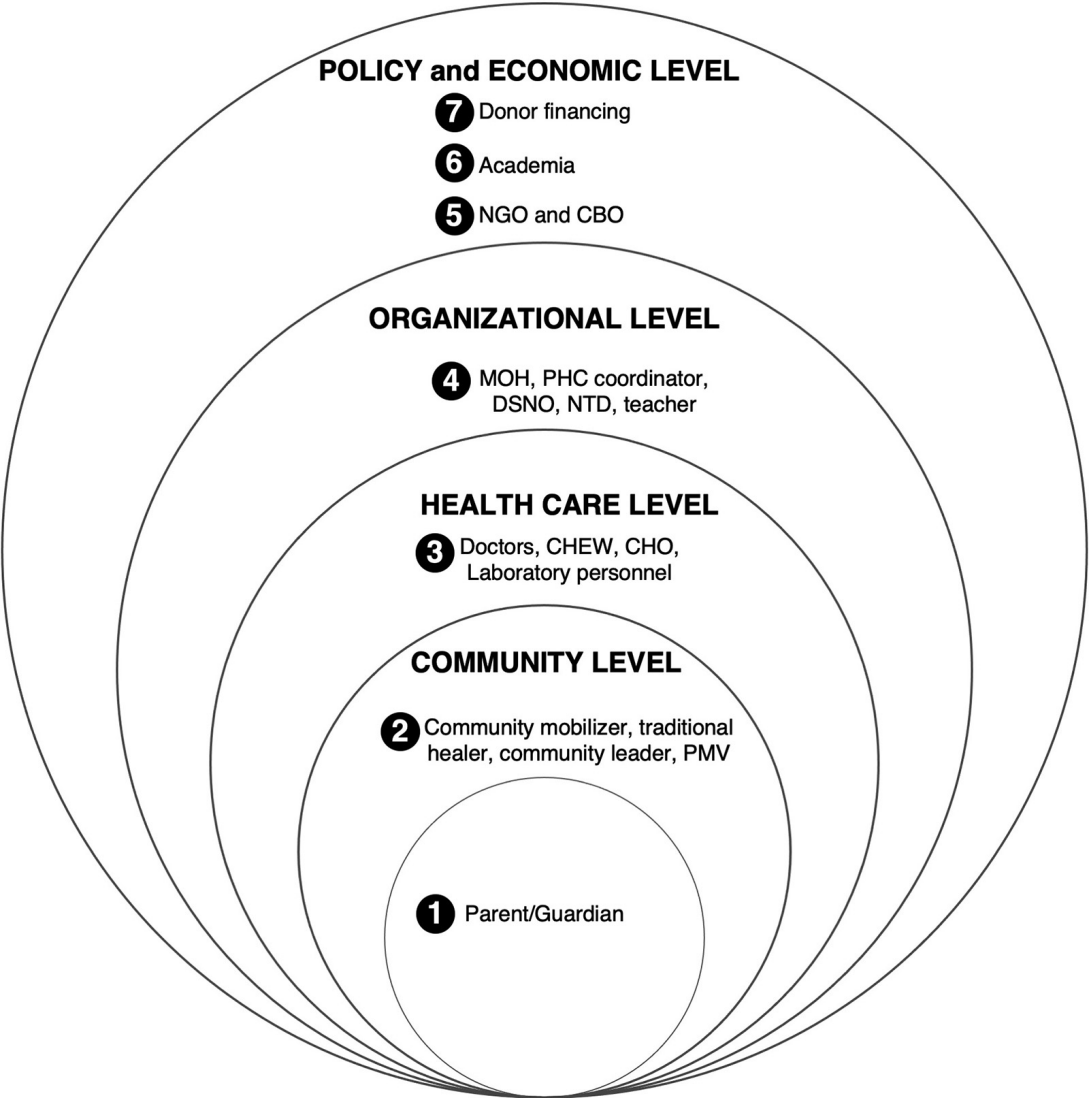
Stakeholders Within the 4 Levels of the Health Care System in Oyo State, Nigeria^5^ Abbreviations: CBO, community-based organization; CHEW, community health extension worker; CHO, community health officer; DSNO, disease surveillance and notification officer; MOH, medical officer of health; NGO, nongovernmental organization; NTD, neglected tropical disease; PHC, primary health care; PMV, patient medicine vendor.

Onasanya et al. identified different stakeholders within 4 levels of the health care system in Oyo that are involved in diagnosing, controlling, and eliminating schistosomiasis:^5^ (1) policy and economic, (2) organizational, (3) health care, and (4) community (Figure 1). Stakeholders at the policy and economic level include financing organizations, nongovernmental organizations (NGOs), and researchers. Organization-level stakeholders are health system managers that are interested in diagnostic devices that streamline the workflow and diagnostic efficiency. These stakeholders include medical officers of health, PHC coordinators, neglected tropical disease (NTD) officers, disease surveillance and notification officers (DSNOs), and teachers. Health care-level stakeholders include doctors, community health officers (CHOs), community health extension workers (CHEWS), and laboratory technicians who are interested in devices, particularly for use within remote areas. Lastly, community-level stakeholders, such as patients and other community members, are interested in diagnosis and treatment but lack expertise in using diagnostic devices for that purpose. Consequently, they rely on health care professionals for diagnoses and treatment.

Even though case management and control and elimination efforts are widely used simultaneously, the different stakeholders follow various procedures in implementing them. For example, at the health care level (level 3 in Figure 1) CHEWs and CHOs prioritize control and elimination at the community level, especially in remote communities that have limited or no health care facilities.^15^^,^^17^^,^^20^^,^^21^ However, doctors and laboratory personnel within the same level prioritize individual case management.

Similarly, the control and elimination approach is complex because it is addressed through a combination of different stakeholder procedures. For example, organizational-level stakeholders (level 4, Figure 1) prioritize screening schools and doing mass drug administration (MDA) to school-aged children in the community or at a cluster of schools, while policy makers (level 5, Figure 1) prioritize MDA on a national level including adults and children.^18^^,^^19^

Even though all these procedures are important in actualizing individual case management and control and elimination, they may require different diagnostic devices that can be applied in different contexts. And these stakeholder groups are interested in different aspects of diagnostic devices. This necessitates new diagnostic devices that can support the various stakeholders in performing tasks targeting schistosomiasis. Therefore, it is important to understand stakeholder preferences and perspectives to reach a consensus on the best strategies for schistosomiasis control.

The main diagnostic tool for urinary schistosomiasis is conventional microscopy, which has critical shortcomings. Conventional microscopy is expensive, laborious to use, depends on well-trained personnel that rural communities often lack, cannot be deployed outside of the lab, and is most appropriate in well-equipped centralized laboratories not found in rural regions where schistosomiasis is prevalent.^1^^,^^3^^,^^9^^,^^22^^–^^24^ As such, potential new diagnostic devices that can support stakeholders in targeting the diagnosis and subsequent control and elimination of urinary schistosomiasis are crucial.

Improved stakeholder capacity to perform such tasks is key to increasing diagnostic coverage, improving control, and eventually eliminating this infection.^25^ However, these goals cannot be achieved if the design of current and new diagnostic devices does not consider the stakeholders’ needs. Therefore, it is crucial to ensure that new diagnostic devices are developed to meet stakeholder needs as a means of supporting them in performing their respective tasks.

To increase diagnostic coverage, improve control, and eventually eliminate schistosomiasis, current and new diagnostic devices need to consider stakeholders’ needs.

Many new diagnostic devices for parasitic infections are under development, ranging from digital optical devices to sophisticated DNA-based analytic devices.^26^^–^^35^ However, these devices are currently not deployable or commercially available and are therefore not available at the point of need.^5^^,^^30^ The World Health Organization estimates that 70% of medical devices in health care facilities in low-resource settings do not function well given that they were designed for the health care context in high-resource countries and were therefore not optimized for other settings.^20^ According to the United to Combat NTDs report on Delivering on Promises and Driving Progress, effective devices for urinary schistosomiasis remain an unmet need.^7^ To be effective, new diagnostic devices for urinary schistosomiasis will need to (1) be designed for specific contexts of use, (2) fit the specific local (health care) infrastructure, (3) incorporate product requirements and performances suited for specific communities, and (4) be commercialized and implemented.^7^^,^^36^

Health care systems are complex and include a diverse number of stakeholder groups, which often bring distinct perspectives on issues related to care delivery and what constitutes appropriate treatment or quality of care.^37^^–^^42^ Taking stakeholder perspectives and specific needs into account will enable them to fulfill tasks and implement strategies targeting the diagnosis and control of urinary schistosomiasis, which will ensure the uptake and use of new diagnostic devices to support the fight against this disease.^35^ Therefore, we aimed to explore and understand the stakeholder perspectives on the context of use and application of potential new diagnostic devices for urinary schistosomiasis in Oyo. Context of use describes the interaction that occurs between the stakeholders (actors), diagnostic device (object), and location, while application describes the action of putting the diagnostic device into operation or use.^36^ In this article, we present the different stakeholder perspectives that were studied.

## METHOD

We applied Q-methodology to elucidate stakeholder perspectives on the context of use and application of new diagnostic devices for urinary schistosomiasis in Oyo. Q-methodology is a technique used in research to investigate subjectivity and people’s viewpoints, perspectives, and beliefs, among other factors, regarding a particular phenomenon.^43^^–^^45^ Q-methodology was suitable for gaining an understanding of the different perspectives on a potential new diagnostic device among the multiplicity of stakeholders who follow different procedures regarding the diagnosis of urinary schistosomiasis. In addition, as mentioned above, the health care system is recognized for its complexities and diverse stakeholder groups with distinct perspectives on issues of care delivery.^37^^–^^42^ Therefore, the use of Q-methodology was suitable in this research to elucidate these different stakeholder perspectives. Q-methodology is used to reveal how and why people think the way they do and to uncover different patterns of thought while relying on a small number of respondents.^46^^,^^47^ Unlike conventional surveys in which participants rate items in a questionnaire, Q-methodology compares perspectives between participants, and through factor analysis, identifies participants who share similar perspectives.^48^^,^^49^

**FIGURE 2:**
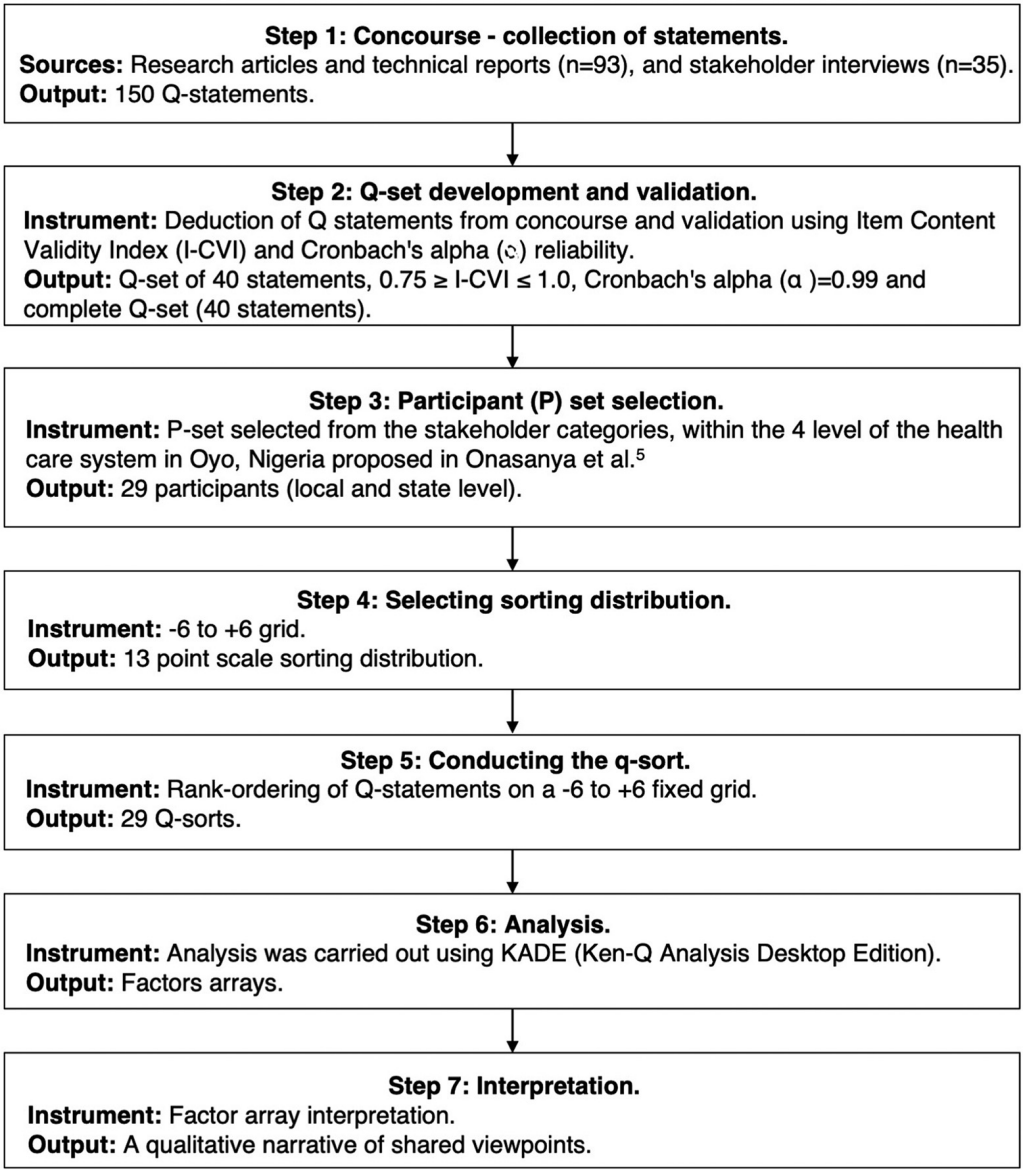
Q-Methodology Study Process Used to Understand Stakeholders’ Perspectives on New Diagnostic Devices for Urinary Schistosomiasis^50^

In this study, the Q-methodology process was guided by the following steps: (1) collection of statements (also known as concourse development); (2) selection, development, and validation of statements deducted from the concourse (also known as Q-set); (3) participant selection (P-set), (4) selecting sorting distribution, (5) conducting the Q-sorting, (6) analysis, and (7) interpretation (Figure 2).^50^ The steps outlined in this study facilitated a step-by-step implementation of Q-methodology as a research tool and did not aim to provide foundational material on how to conduct Q-methodology. Previous publications can be used to gain an in-depth understanding of Q-methodology.^39^^,^^50^^,^^51^

### Step 1. Collection of Statements (Concourse)

A collection of 150 statements (Supplement 1) were gathered from literature (n=93) and stakeholder interviews (n=35) to build the concourse. Statements from literature were sourced from journal articles, conference proceedings, and NGO and government reports that contained insights on diagnosing urinary schistosomiasis in Nigeria and sub-Saharan Africa.^52^ A variety of terms and combinations were searched in academic databases such as PubMed, Science Direct, and Google Scholar. The following search terms were combined using the logical operators “and” or “or”: schistosomiasis diagnostic, tools, schistosomiasis stakeholder involvement, Nigeria, application, context of use, diagnostic tools for *S. haematobium*, approaches, and strategies. Statements pertaining to diagnosis, treatment, and patient-related diagnostic needs were identified by interviewing stakeholders from the 4 levels of the health care system (Table 1). These interviews provided additional opinion statements, which were combined with the findings from the literature.

**TABLE 1.** Stakeholders Interviewed for Perspectives on the Context of Use and Application of Potential New Devices to Diagnose Urinary Schistosomiasis, Oyo, Nigeria^5^

**Health Care Level**	**Stakeholder**	**Interview Count **
Policy	Nongovernmental organization	1
	Academia/researcher	2
Organizational	Primary health care coordinator	1
	Medical officer of health	1
	Disease surveillance notification officer	2
	Neglected tropical disease officer	3
	Teacher	6
Health care	Doctors	1
	Community health extension worker	4
	Laboratory technician	4
	Community health worker	2
Community	Patient/guardian	5
	Community mobilizer	1
	Traditional healer	1
	Community leader	1

### Step 2. Q-Set Development and Validation

A Q-set is a selection of statements deducted from a concourse (step 1).^44^^,^^51^^,^^53^ Forty statements were selected from the concourse in step 1 to make up the Q-set^53^ (Supplement 2 Table 1). A Q-set of 40 opinion statements provided good coverage of the study, was sufficient to elicit existing viewpoints, and fell within the sample range (i.e., 30–50) that is generally accepted in Q-methodology.^43^^,^^51^^,^^54^ The statements were selected based on 4 considerations as proposed by Uniting to Combat NTDs target product profiles, which provided a theoretical framework and ensured that the Q-set covered the essential diagnostic product requirements for diagnosing urinary schistosomiasis. Specifically, these requirements included (1) context use case, (2) infrastructure, (3) product requirements (design and performance), and (4) rollout strategy.^7^^,^^36^^,^^55^ Infrastructure describes the facility, location, or setting, and product requirement describes the specifications for device design and performance. Rollout strategy describes strategies to introduce, integrate, and commercialize a new product to users. Four domain experts validated the 40 selected statements. Three experts in the domain of parasitology and schistosomiasis diagnostics provided validation regarding urinary schistosomiasis diagnosis within the Nigerian context, and 1 Q-methodology expert provided validation on the construction of the Q-set. The domain expert validations were aimed at measuring the internal consistency and content validity of the Q-set. The internal consistency of the Q-set was measured using Cronbach’s alpha (α) reliability coefficient and the content validity was measured using the Item Content Validity Index (I-CVI).^56^

In measuring the content reliability and validity, the experts rated each of the Q-statements for readability, clarity of statement, and heterogeneity (breadth and depth)^53^ (Supplement 2 Table 2.) Every statement was clear and made its own original contribution to the Q-set, without overlaps.^51^ The domain expert rating (Supplement 3) was used to compute a statistical analysis of Cronbach’s alpha reliability coefficient using Windows SPSS 26. The resulting Cronbach’s alpha reliability coefficient was 0.99, which is acceptable and higher than the Nunnally norm of 0.70^57^ (Supplement 4). Similarly, the expert ratings were used to compute an I-CVI value for each of the 40 statements (Supplement 3): 32 statements scored an I-CVI value of 1.00, 8 statements had an I-CVI value of 0.88, and 1 statement had an I-CVI score of less than 0.80. Experts suggested minor changes in statement wording to improve clarity and readability, especially for the Q-statements with I-CVI values of less than 0.80.^56^ As a result of expert feedback, minor edits were made to 2 statements (including statements with I-CVI values greater than 0.80) and 38 statements remained unchanged (Supplement 2 Table 1).

### Step 3. P-Set Selection

A total of 29 participants were purposively selected from 3 of the 4 health care system levels in Oyo (Table 2). We selected individuals based on their medical and scientific expertise in urinary schistosomiasis diagnosis and treatment in Oyo,^5^ as well as their willingness to participate. Participants, which included individuals from the policy and economic, organization, and health care levels, represented high power and interest in the adoption of new diagnostic devices for schistosomiasis in Nigeria. We did not include community-level participants because they lacked the expertise in diagnosing urinary schistosomiasis using diagnostic devices and relied on health care professionals for diagnoses and treatment. The selected participants (n=29) were sufficient to establish and compare the different perspectives expressed in the Q-set. Likewise, a Q-set larger than the participant number was sufficient for this study because of the relevant background of the participants.^51^

**TABLE 2.** Participant-Set Composition for Perspectives on the Context of Use and Application of Potential New Devices to Diagnose Urinary Schistosomiasis, Oyo, Nigeria

**Stakeholder Level**	**Stakeholder**	**No. of Participants**	
		**Local Level**	**State Level **
Policy and economy	Nongovernmental organization	1	1
	Financing	—	2
	Academia/researcher	1	1
Organizational	Medical officer of health/primary health care coordinator	1	1
	Disease surveillance notification officer	1	1
	Neglected tropical disease officer	2	1
Health care	Medical doctor	1	2
	Community health extension worker	1	1
	Laboratory technician	4	2
	Community health officer	4	1

### Step 4. Selecting a Sorting Distribution

**FIGURE 3:**
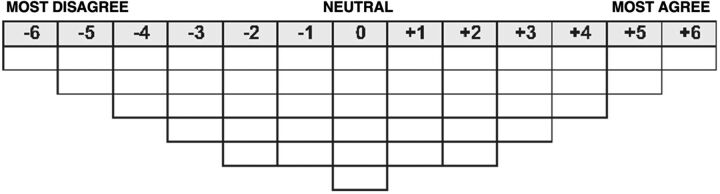
Flatten-Gaussian 13-Point Scale Sorting Fixed Distribution

Data collection involved participants’ rank-ordering statements on a fixed distribution. Compared with a free distribution, a fixed sorting distribution created an opportunity to standardize the process of ranking the statements.^51^ Within this study, a Flatten-Gaussian 13-point scale (−6 to +6) sorting fixed distribution with the poles labeled “most agree” to “most disagree” (Figure 3) was selected. This shape was selected to maximize the participant’s proficient knowledge of the topic in achieving a granular rank order,^51^ regarding the most relevant context and application of use for a new urinary schistosomiasis diagnostic device in Oyo.

### Step 5. Conducting the Q-sort

Q-sort involved rank-ordering the 40 statements on the Flatten-Gaussian 13-point scale sorting distribution grid. A total of 29 Q-sorts were conducted within this research. Q-sorts were conducted using Easy-HtmlQ 2.0; a web-based platform for online Q-administration.^58^ Easy-HtmlQ 2.0 was used to administer Q-sort remotely while providing live support via secure online calls within the boundaries of the COVID-19 pandemic. Conducting this study remotely or virtually, while not ideal, is an acceptable approach in Q-methodology.^59^^–^^61^ The Easy-HtmlQ user-friendly interface allowed participants to familiarize themselves with the Q-set on digital cards. To reduce cognitive load, participants were first allowed to organize the statements into 3 piles: “strongly agree,” “strongly disagree,” and “neutral.” Secondly, the 3 piles were ranked on the sorting grid from “most agree” (+6) to “most disagree” (−6). Each Q-sort exercise was completed with post-sorting questions within the Easy-HtmlQ 2.0 to elucidate the reasons why statement rankings fell in extreme corners of the sorting grid and to understand the reasoning underlying rankings and trade-offs. The steps of sorting and post-sorting questions are in line with recommended practices in Q-methodology.^44^^,^^62^ Each Q-sorting lasted approximately 35–45 minutes per participant.

The study was conducted with ethical approval from the Research Ethics Committees at the University of Ibadan-Nigeria (REF UI/EC/21/0100) and Delft University of Technology, the Netherlands. Participants were assured that participating in the study was voluntary and that information collected was anonymized and treated with confidentiality. All participants signed and provided informed consent before participating in the study and were allowed to withdraw from the study at any time. No participants expressed any hesitation to participate in the study.

### Step 6. Analysis

Analysis was carried out using KADE (Ken-Q Analysis Desktop Edition) version 1.2.1 program.^63^^,^^64^ The KADE program provided a simple and interactive visualization to analyze and interpret the data gathered. Q-sorts were entered into the KADE program for intercorrelation, and factors, also known as common perspectives, were identified using centroid factor analysis and varimax rotation. Fundamentally, factor analysis identifies the patterns of relationships within the data, or in this case participants’ perspectives, and thereafter summarizes them into distinct patterns of occurrence.^50^^,^^65^ Centroid factor analysis was preferred in this study because of its permissiveness for data exploration as opposed to principal component analysis, which resolves the data into a single mathematically best solution.^51^ Varimax was preferred because it provides the most mathematically preferred solution in generating factors that when put together account for the maximum amount of study variance.^51^ Varimax procedure in the KADE program provided factor loadings sorted from highest to lowest.^63^

Factor loadings sorted from highest to lowest provided a solution towards working our way down from the strongest factor load accounting for the maximum amount of study variance and produced clear and consistent factor interpretation and clustering.^51^ Similarly, factors were retained for extraction if they had an eigenvalue (EV) ≥ 1.00 and 2 or more significant loadings (0.05 significant) following extraction.^51^^,^^54^ EV is an indication of a factor’s statistical strength. Factors with an EV less than 1.00 are often taken as a cutoff point, and factors with an EV above 1.00 are important and retained for extraction.^51^ Iteration of the factor retention and extraction process continued until clear and consistent factor explanations and clustering emerged. At the end of this iterative process, the retained factors were documented in factor array scores and crib sheets (Supplements 5 and 6). A factor array table is a configuration of a Q-sort showing the viewpoint of a particular factor, grouping of statements, and their specific ranking value.^51^ Crib sheet is a tool designed by Simon Watts of Nottingham Trent University, and it is used to examine factor array in detail through a systematic and methodical approach that is consistent and delivers a holistic factor interpretation.^51^^,^^66^

### Step 7. Interpretation

The factor array scores and crib sheets were interpreted to produce factor themes. Factor arrays provided the best possible estimates of relevant and holistic viewpoints and crib sheets were used to enforce holism by forcing engagement with every item in a factor array.^51^ Statements with a statistical significance (*P*≤.05) were considered distinguishing statements.^43^^,^^54^ The results and interpretation of the analysis were summarized in a qualitatively rich narrative with a coherent overview of different perspectives (factors), its element, and the line of reasoning. For factor interpretation, it should be noted that the statement number and its corresponding rank in the factor array are represented as [statement; score]. For example, [24; −1] means statement 24 is ranked at −1 along the sorting grid (−6 to +6). This system of interpretation produces a succinct and holistic narrative because it shows how statements are linked within a factor.^67^ The summarized narratives of the factors were further validated by experts that were representative of participants in each factor narrative. All experts had either medical or scientific expertise in diagnosing urinary schistosomiasis in Oyo, Nigeria. Each expert was assigned to read the factor they represented and engaged in qualitative discussions on whether the narrative was representative of the perspective shared within the diagnostic landscape in Oyo or not.

## RESULTS

To understand the stakeholder perspectives on the context of use and application of new diagnostic devices for urinary schistosomiasis in Oyo, Nigeria, a by-person factor analysis of 29 Q-sorts was performed. A factor array score of 4 distinct factors emerged (Table 3). These 4 factors collectively explained 33% of the study variance. A total explained variance of 33% is less than the widely used minimum range of 35%–40%.^68^ However, a low explained variance is not necessarily problematic and may be meaningless, especially when great care has been taken in selecting the Q-set and participant set.^51^^,^^54^^,^^69^ Having only mathematical solutions such as total explained variance limits the opportunity to engage with the ranked statement.^51^ Notably, a 5-factor solution would have offered a higher explained variance; however, it produced unclear and inconsistent factor explanation and clustering generated in the crib sheet (Supplement 5). A concise and consistent interpretation and explanation of factors or viewpoints were best achieved with a 4-factor solution (Supplement 6).

**TABLE 3.** The 40 Statements and the Factor Array Scores of the 4 Factors

**No Statement**		**Factors**				
		**F1**	**F2**	**F3+**	**F3−**	**F4**
	**Context (Use Case)**					
1	Microscopy using a concentration technique is the recommended method to prove active schistosomiasis, despite its low sensitivity and need for expert users.	0	−1	−1	−2	−3
2	Diagnosis should include the identification of *Schistosoma* parasites in both humans and water sources that may be contaminated.	+2	0	+4	+2	0
3	Mass screening and diagnosis should be carried out alongside mass drug administration with praziquantel.	−2	−3	+4	−2	5
4	Schistosomiasis surveillance enables program managers to monitor the effectiveness of intervention strategies and identify which populations require continuing interventions.	−1	+5	+2	−1	+3
5	The availability of RDTs, which requires only minimal infrastructure, would improve diagnosis and surveillance simultaneously.	+3	−2^b^	+3	+2	+5
6	Implementing an affordable and simple POC diagnostics solution will reduce the financial burden of equipment and personnel at each health facility.	0^b^	−2	−2	4^b^	−1
7	POC diagnostics that can detect and confirm cases immediately will reduce the risk of missed or misdiagnosed cases.	+2	0	−3^a^	0	1
8	The quantification of egg excretion helps to assess the transmission potential of populations living in endemic areas.	−1	−2	1	−1	−2
9	Schistosomiasis control programs should target school-aged children only.	−6	−6	−1^b^	−5	−6
10	Due to the low level of education and lack of training among community health workers, incorrect treatment is often prescribed.	−3^a^	−1	0	0	3^b^
11	Presenting data on the severity of schistosomiasis infection of specific locations will guide the development of strategies for effective case management and control elimination.	+1	+2	−6^b^	+3	+1
12	Passive case detection, based on people’s self-reporting, has been considered a less expensive strategy for the control of schistosomiasis.	−3	−2	6^b^	0	0
13	Prevalence and intensity of infection is often higher among children than among adults.	0	−1	−3	+1	0
	**Infrastructure and location**					
14	Schistosomiasis diagnosis should be done closest to the community as it reduces the time to carry samples back to the laboratory.	0	+1	2	0	+4
15	Diagnostic and treatment campaigns should target school-age children, adolescents and those whose occupations involve contact with infectious water (e.g. fishing, farming, irrigation, and domestic tasks in water).	−2	+3	+1	+1	−1
16	Simple, rapid POC tests should be used in primary health care settings where patients often travel long distances to access health care facilities.	−1	−1	−2	+2	+2
17	Diagnostic devices should be deployed in primary health care centers, clinics, and health posts since they are the most lacking in equipment.	−4^a^	+4	−1^a^	+5	1^a^
18	Testing of urine samples for schistosomiasis with school-based surveys should be done at the school location.	−1	+1	0	−2	−5
19	It is convenient to treat patients for schistosomiasis infection without a confirmed diagnosis due to the delay in receiving test results from referral hospitals.	−5	−5	−5	−4	−4
	**Product requirement**					
20	Schistosomiasis elimination calls for developing novel diagnostic tools with higher sensitivity and specificity than microscopes.	+1	3^b^	−4	−1	−3
21	Diagnostic device for schistosomiasis with minimal to no sample preparation is ideal.	−2	−3	−2	−3	−5^a^
22	The diagnostic device should quantify eggs to provide an estimation of the number of people that have been exposed to schistosomiasis in a population.	5^a^	−5^b^	+1	−1^a^	+2
23	Devices should be easy to use by medical personnel and health workers such as CHEWs, CHOs, laboratory scientists to detect and diagnose schistosomiasis-infected patients.	+1	+2	0	−5	−2
24	Patient samples should be processed in batches to get a faster turnaround time and increase the efficiency of sample processing during mass campaigns or sensitization meetings.	0	0	+1	−4^b^	+3
25	Ideal diagnostic approaches should allow the concurrent detection of several pathogens in different biological samples such as urine, blood, and stool.	+3	0	+3	−3^a^	+1
26	Diagnostic devices should be sensitive enough for detecting very light schistosomiasis infections.	+4	−3	+5	−1	−1
27	Diagnostic devices should have their own reliable power sources due to the unstable power connectivity in rural and distant communities.	+6	−1	−1	+6	+6
28	The best diagnostic devices should be easy to transport safely by car, motorbike, and bicycle to remote locations.	4^a^	+1	−4	+2	−1
29	Diagnostic devices should be compact and portable so that they can be easily deployed in the community.	+2	0^a^	+3	+3	−3^b^
30	Diagnostic devices/tests should identify and map out areas with a large spread of schistosomiasis and be able to trace the source of the disease.	3a	0	0	0	−1
31	Devices should be locally repaired and maintained by local technicians in case of breakdown.	0	+2	−5^b^	+4	0
32	The device should be easy to clean and disinfect to prevent re-contamination.	+1	+1	0	+1	+2
	**Rollout strategy**					
33	The cost per diagnostic test should be free (covered by the government).	−1	4a	0	+1	−2
34	Cost per diagnostic test should be less than 1,000 Naira (US$2).	−5^b^	6^a^	−3	+3^a^	−2
35	Mass drug administration campaigns should be accompanied by mass diagnostic and disease awareness campaigns.	−2	+2	+2	1	0
36	Data from diagnostic devices should be accessible to stakeholders (local government, DSNO, MOH, researchers, and NGOs) to enhance planning.	+1	+5	+2	0	+2
37	New interventions should consider training the health care workers at the community level and the informal sector (PMVs and traditional medicine) to increase coverage to diagnostics.	−3	+1	−1	−6^b^	0
38	Diagnostic tools for schistosomiasis should be deployed and used at the community level by PMVs and community mobilizers as they already serve as trusted stakeholders in the community.	−4	−4	1^b^	−3	−4
39	The role of the village/community head is important in the acceptance of the new diagnostic device.	+5	−4^a^	−2^a^	+5	1^b^
40	Patients with schistosomiasis should be tested before being treated.	+2	+3	+5	−2^b^	+4

Abbreviations: CHEW, community health extension worker; CHO, community health officer; DSNO, disease surveillance notification officer; MOH, medical officer of health; NGO, nongovernmental organization; PMV, patent medicine vendor; POC, point-of-care; RDT, rapid diagnostic test.

a Distinguishing statement significant at *P*<.05.

b Distinguishing statement significant at *P*<.01.

The interpretation of the factors revealed that the context of use and the application of new diagnostic devices for urinary schistosomiasis are both strongly influenced by 4 distinct factor themes. That is, new diagnostic devices will need to (1) be deployable to remote or distant communities, (2) be affordable, (3) identify and confirm infection status before treatment in patients with a diagnosis of urinary schistosomiasis based on self-reporting and be optimized for the local setting, and (4) fit within local minimal infrastructural settings (Table 4).

**TABLE 4.** The 4 Distinct Factor Themes That Emerged From the Stakeholder Perspective on the Context of Use and Application of a Potential New Diagnostic Device for Urinary Schistosomiasis in Oyo, Nigeria

**Factors**	**No. of Sorts**	**Loaders**	**Factor Theme**
Factor 1	10	2 laboratory technicians, 2 donor financing, an NGO representative, a researcher, DSNO, MOH, medical doctor, and a CHEW	Deployable diagnostic devices to remote/distant communities
Factor 2	5	2 lab technicians, a CHEW, researcher, and a DSNO	Affordable diagnostic tests/devices
Factor 3+	2	2 CHOs	Identify and confirm infection status before treatment in patients with a diagnosis of urinary schistosomiasis based on self-reporting
Factor 3–	2	A medical doctor and a laboratory technician	Equip health care facilities with diagnostic devices optimized for the local setting
Factor 4	5	An NTD officer at state level, a medical doctor, a CHEW, a CHO, and an NTD officer in an LGA	Simple POC devices/tests requiring minimal local infrastructure
Confounded	3		
Nonsignificant	2		
Total	29		

Abbreviations: CHEW, community health extension worker; CHO, community health officer; DSNO, disease surveillance notification officer; LGA, local government area; MOH, medical officer of health; NGO, nongovernmental organization; NTD, neglected tropical disease; POC, point-of-care.

### Factor 1: Deployable Diagnostics Devices to Remote or Distant Communities

Factor 1 (F1) had an EV of 4.77 and explained 16% of the total variance. A total of 10 respondents loaded significantly in this factor, and all in the positive pole. All 10 respondents were decision makers and performed supervisory, financing, and technical roles targeting the diagnosis of urinary schistosomiasis in Oyo. Four of the 10 respondents in F1 represented the policy and economic level of the health care system, including 2 individuals in the donor financing sector, 1 schistosomiasis-control coordinator within the NGO sector, and 1 state university researcher. F1 also involved a DSNO and a medical officer of health at the state level (federal government). They represented the organizational level of the health care system that focuses on the pragmatic parts of schistosomiasis control, such as gathering information about urinary schistosomiasis that is then used for program planning. Similarly, F1 included 2 laboratory technologists, 1 medical doctor, and 1 CHEW at a state-run PHC facility. These participants represented the health care level that is interested in devices that improve the speed and accuracy of diagnosing urinary schistosomiasis.

**FIGURE 4:**
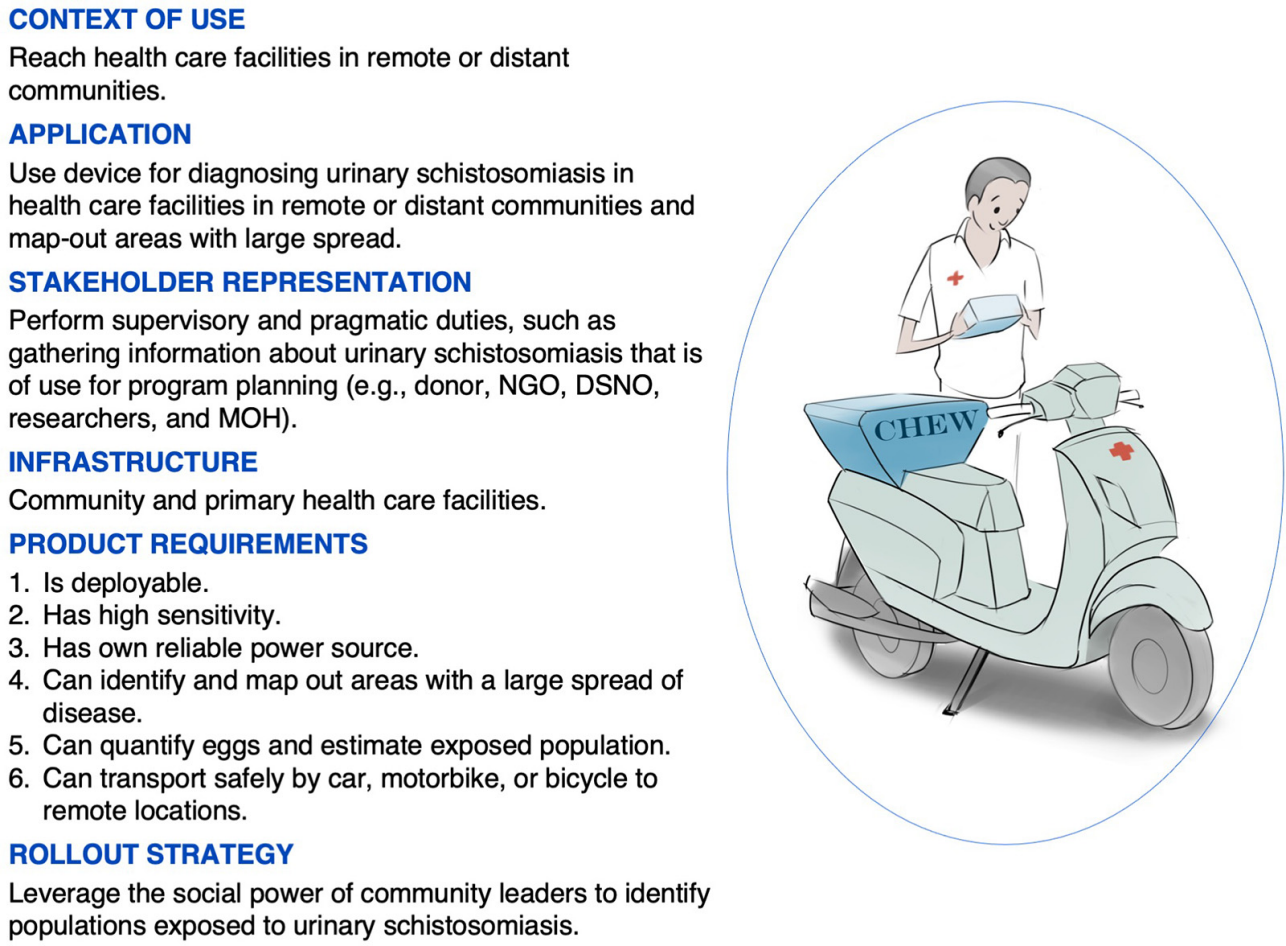
Factor 1: Context of Use and Application of a Deployable Diagnostic Device for Diagnosing Urinary Schistosomiasis in Ibadan, Nigeria Abbreviations: DSNO, disease surveillance notification officer; MOH, medical officer of health; NGO, nongovernmental organization.

F1 respondents emphasized the need for new diagnostic devices for urinary schistosomiasis that are deployable to remote communities and health care facilities [29; +2, 17; −4] (*P*≤.05) (Figure 4). New diagnostic devices that are deployable to remote communities will require their own reliable power sources owing to limited power connectivity in such areas [27; +6]. Similarly, deploying these new diagnostic devices to remote communities and health care facilities should be possible using bicycles, motorbikes, and cars [28; +4] (*P*≤.05). This will increase the ability to reach remote communities to detect *S. haematobium* cases and map areas with a large spread of the infection [30; +3*] (*P*≤.05). Deployable diagnostic devices will need to be sensitive and able to quantify eggs to estimate the intensity of infection [22; +5] (*P*≤.05). Estimating the population exposed to urinary schistosomiasis should not be limited to school-age children but should also include adults [9; −6]. The ability to identify populations exposed to this infection could be enhanced by leveraging the social power of community leaders [39; +5]. However, F1 respondents raised concerns about including nonclinical professionals in diagnostic processes [37; −3, 38; −4].

### Factor 2: Affordable Diagnostic Tests and Devices

Factor 2 (F2) had an EV of 1.88 and accounted for 6% of the total variance. Five participants loaded significantly in the positive pole: 2 PHC laboratory technicians, 1 CHEW, 1 researcher, and 1 DSNO. All 5 participants operated in local government areas (LGAs) in Oyo. The PHC laboratory technician and CHO were interested in new devices that improve the speed and accuracy of diagnostics. They were proactive in the community and demonstrated high social relationships with the community members. The researcher represented the policy and economy level and provided technical expertise targeting the control and elimination of schistosomiasis at LGAs. The DSNO was interested in gathering and using prevalence data for planning.

**FIGURE 5:**
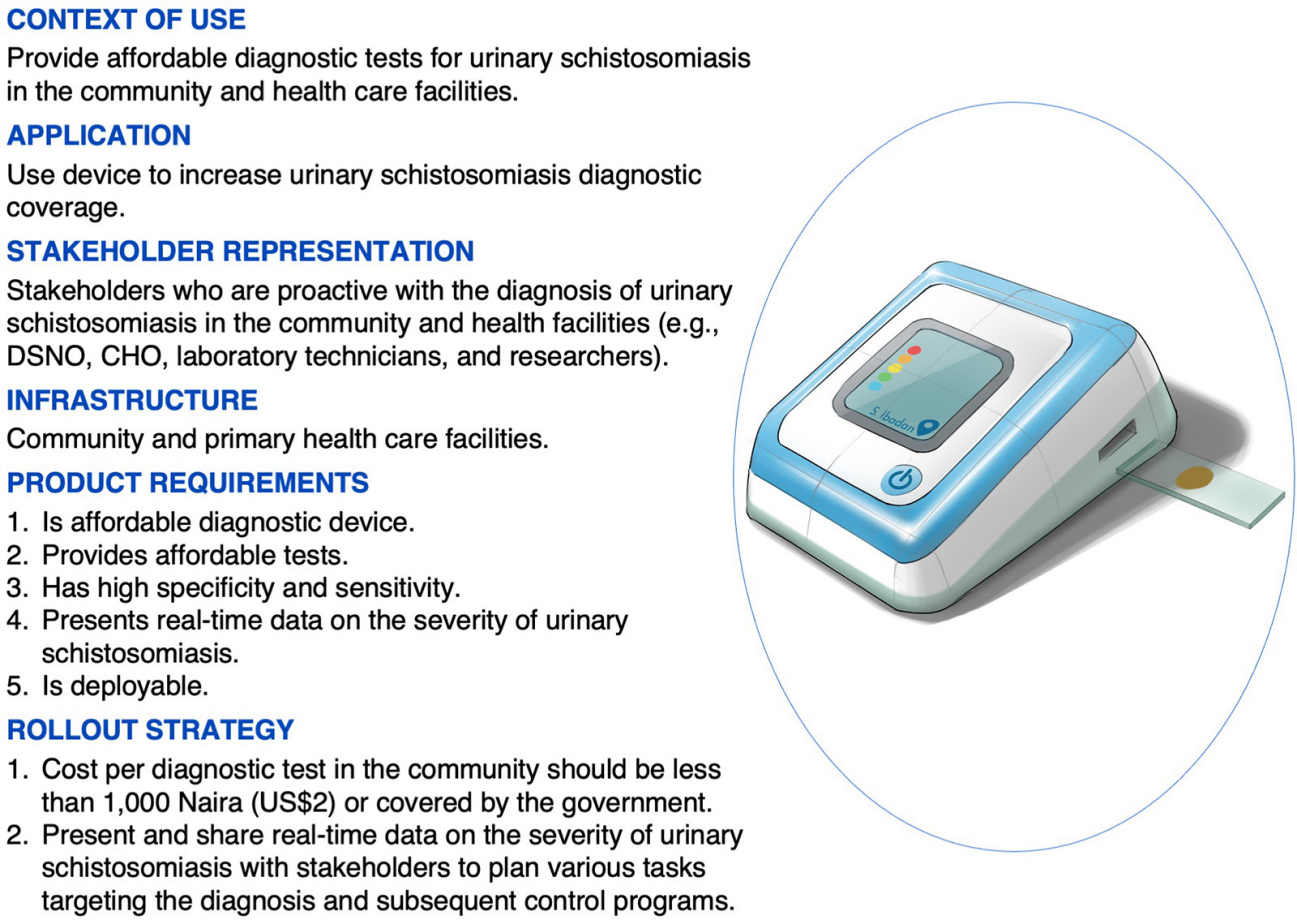
Factor 2: Context of Use and Application of Affordable Diagnostic Tests/Devices for Diagnosing Urinary Schistosomiasis in Oyo State, Nigeria Abbreviations: CHO, community health officer; DSNO, disease surveillance notification officer.

F2 respondents emphasized that the cost per diagnostic test in the community should be less than 1,000 naira (₦), approximately US$2, to be considered affordable or be covered by the government [33; +4, 34; +6] (*P*≤.05) (Figure 5). Affordable or free diagnostic tests will help increase diagnostic coverage in the communities and improve the ability to identify infected areas in need of treatment, MDA, or disease awareness campaigns [35; +2, 19; −5, 3; −3]. Diagnostic tests and treatment campaigns should not only target school-age children [9, −6] but should include other high-risk groups such as adolescents and people whose occupations involve contact with infectious water [15; +3].

Affordable or free diagnostic tests will help increase diagnostic coverage in the communities and improve the ability to identify infected areas in need of treatment.

Respondents in F2 further emphasized developing novel diagnostic devices that are affordable and deployable, have high sensitivity and specificity, and present (real-time) data on the severity of urinary schistosomiasis [20; +3, 11; +2] (*P*≤.01). Having data on infection severity will enable stakeholders to plan various tasks targeting the diagnosis and subsequent control of urinary schistosomiasis [36; +5, 4; +5]. These new diagnostic devices should be deployed in communities and PHC facilities that are most lacking in general laboratory equipment [17; +4, 23; +2, 38; −4].

### Factor 3: Identify and Confirm Infection Status Before Treatment

**FIGURE 6:**
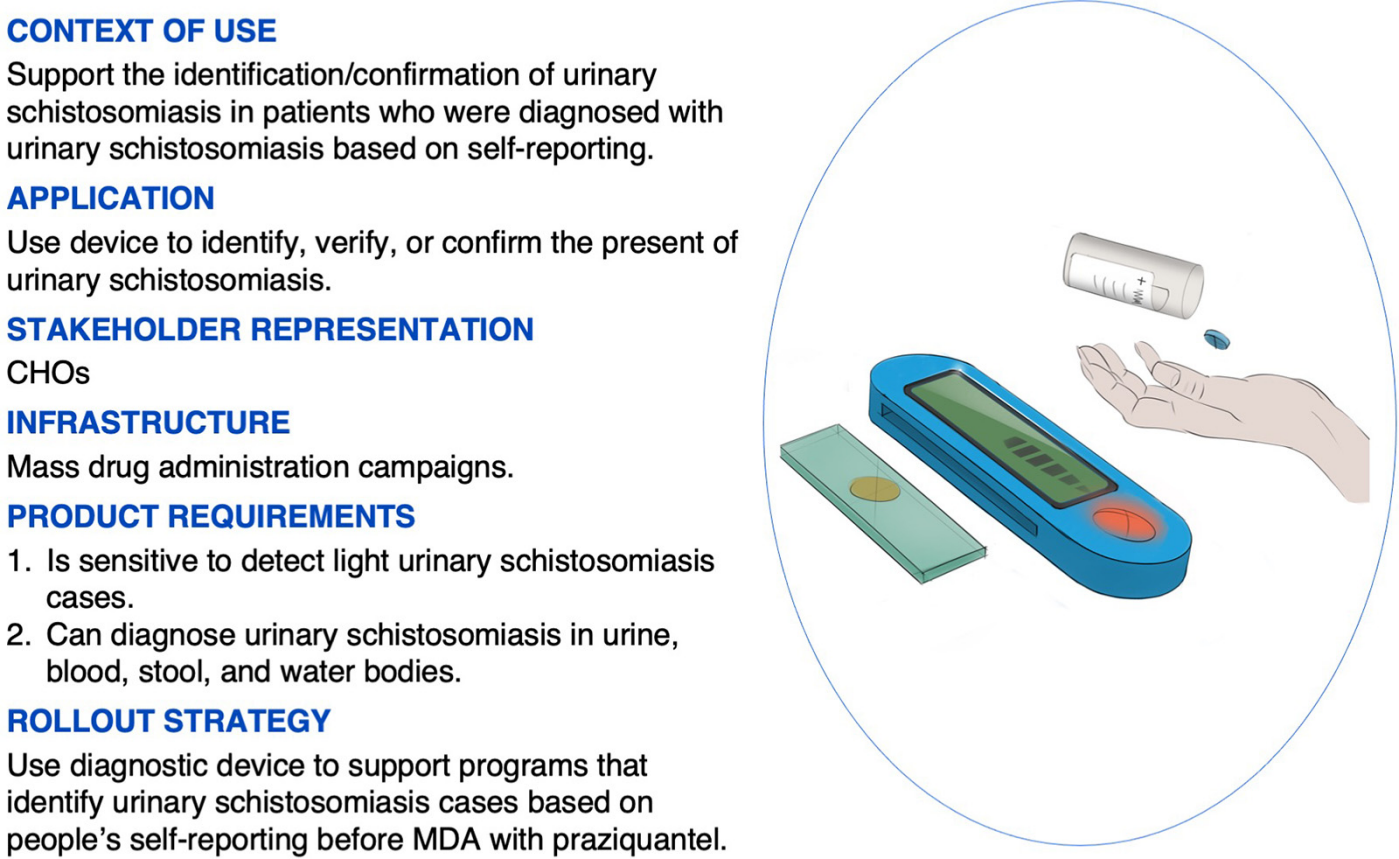
Factor 3+: Context of Use and Application of a New Diagnostic Device for Diagnosing Urinary Schistosomiasis in Oyo State to Identify Disease Status Before Treatment Abbreviations: CHOs, community health officers; MDA, mass drug administration.

Factor 3 (F3) has an EV of 1.65 and accounts for 6% of the total variance. Four participants loaded significantly in this bipolar factor. Two in the positive pole (F3+) and 2 in the negative pole (F3−). The F3+ respondents were both CHOs interested in a new diagnostic device increasing the speed and accuracy in identifying urinary schistosomiasis cases in the LGAs. Respondents in F3− included a laboratory technician at a private laboratory and a medical doctor at a tertiary health facility that offers specialized health care in the form of community-based outreach. F3+ respondents confirmed that passive case identification based on people’s self-reporting is an affordable strategy to identify urinary schistosomiasis [12; +6] (*P*≤.01). (Figure 6). However, case identification based on people’s self-reporting should be supported with a diagnostic device that can identify and/or confirm infection status [3; +4] to support treatment with praziquantel [40; +5, 19; −5]. Diagnostic devices that can quickly identify infection status before treatment should be sensitive to detect a low-level infection [26; +5, 25; +3].

Case identification based on people’s self-reporting is affordable but should be supported with a diagnostic device that can identify and/or confirm infection status.

**FIGURE 7:**
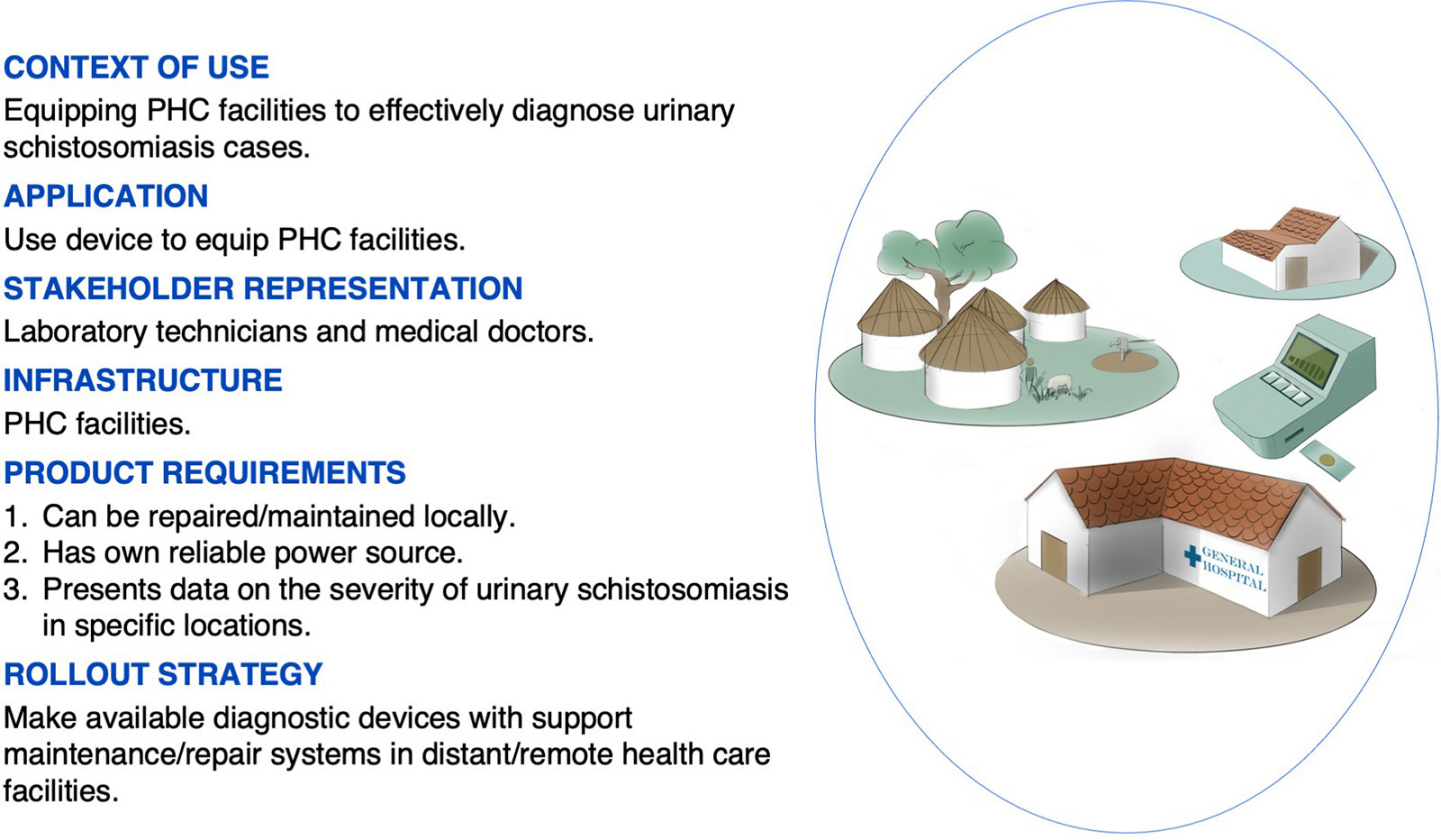
Factor 3: Context of Use and Application of a New Diagnostic Device for Diagnosing Urinary Schistosomiasis in Oyo State to Equip Primary Health Care Facilities Abbreviation: PHC, primary health care.

Similar to F3+ respondents, F3− respondents did not emphasize the need to identify infection status before treatment [40; −2] (*P*≤.01). F3− respondents emphasized the need to detect and obtain data on the severity of infections in multiple biological samples [25; −3, 11; 3] to increase the capacity to identify infected groups and areas. Similarly, such identification will guide the development of strategies to manage and control schistosomiasis within such groups and areas. Community leaders who have high social power can support strategies to manage and control schistosomiasis in infected areas [39; 5]. However, F3− respondents raised concerns about training community members with no formal clinical expertise to detect and diagnose schistosomiasis [23; −5] [37; −6] (*P*≤.01). F3− respondents also emphasized equipping PHC facilities that currently have limited or no diagnostic devices to detect *S. haematobium* [17; +5, 16; +2] (Figure 7). New diagnostic devices that equip PHC facilities to detect *S. haematobium* will need to be maintained and repaired locally and to have their own reliable power sources owing to limited power access in some remote communities [27; +6, 31; +4, 29; +3].

### Factor 4: Simple Point-of-Care Devices/Tests Requiring Minimal Local Infrastructure

Factor 4 (F4) has an EV of 1.35 and accounts for 5% of the total variance. Five participants loaded significantly in the positive pole. That is, NTD officers and medical doctors providing supervisory activities targeting urinary schistosomiasis in multiple LGAs. A CHEW and a CHO stationed at a community PHC facility were also included. F4 respondents were interested in new diagnostic devices that can improve the speed and accuracy of detecting *S. haematobium* cases in PHC facilities.

**FIGURE 8:**
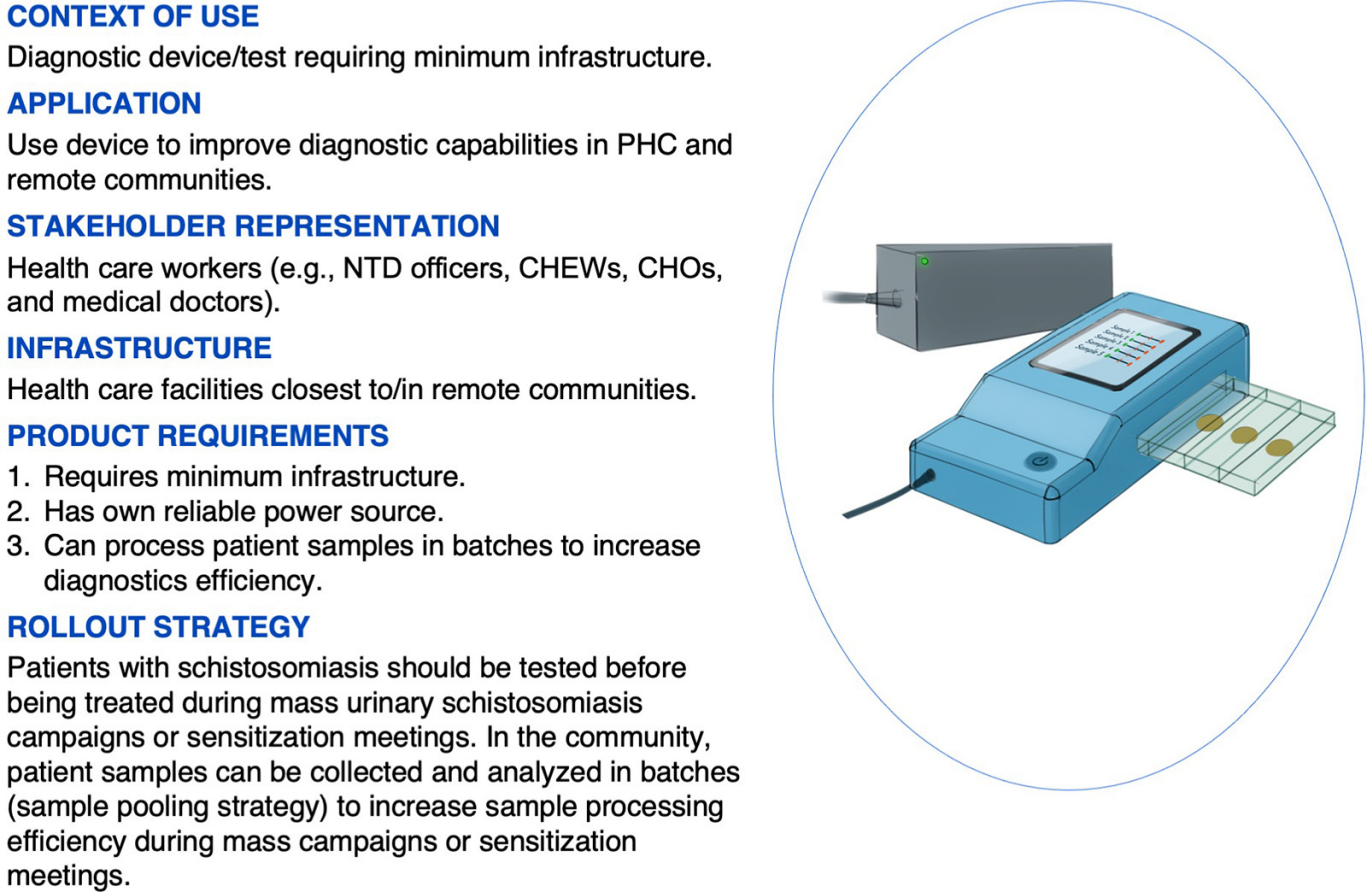
Factor 4: Context of Use and Application of a Simple POC Diagnostic Device for Diagnosing Urinary Schistosomiasis in Oyo State, Requiring Minimal Infrastructure Abbreviations: CHEW, community health extension worker; CHO, community health officer; NTD, neglected tropical disease; PHC, primary health care; POC, point-of-care.

F4 respondents emphasized the need for simple point-of-care (POC) diagnostic devices or tests that require minimal infrastructure [5; +5, 16; +2] (Figure 8) where patients often travel long distances to access health care facilities [16; +2]. Meeting this need will improve diagnostic coverage and surveillance beyond school-age children [3; +5, 16; +2, 9; −6, 18; −5, 5; +5]. Similarly, implementing simple POC diagnostic devices will ensure that the diagnosis is performed closest to the community [14; +4]. In the community, patient samples can be collected and analyzed in batches (sample pooling strategy) to increase sample processing efficiency during campaigns or sensitization meetings [24; +3, 40; +4]. This approach will reduce the time needed to analyze individual patient samples in centralized laboratories, which are often far away [14; +4]. Simple POC diagnostic devices or tests that require minimal infrastructure would require their own reliable power sources, especially in remote communities with limited power access [27; +6].

F4 respondents emphasized the need for simple POC diagnostic devices or tests that require minimal infrastructure where patients often travel long distances to health care facilities.

### Validation of Results

To validate the results of this study, qualitative discussions were carried out with 5 experts about the 4 factors that emerged on the context of use and application of new diagnostic devices in Oyo, Nigeria. The experts included a laboratory technician, a researcher, a CHO, a medical doctor, and an NTD officer. Each expert was representative of loaders in each of the 4-factor themes (Table 4). The experts mentioned that all 4 factors were representative of viewpoints shared within the diagnostic landscape in Oyo. Interestingly, the viewpoints expressed in each factor were closely directed towards improving the ability of stakeholders to perform various tasks targeting the diagnosis and subsequent control of schistosomiasis. Such tasks included identifying and gathering information on cases or areas that are affected by urinary schistosomiasis in Oyo. This finding suggests that the stakeholder perspectives on the context of use and application of new diagnostic devices are closely associated with the different stakeholder tasks and strategies targeting urinary schistosomiasis in Oyo.

## DISCUSSION

This study explored stakeholder perspectives on the context of use and application of potential new diagnostic devices for urinary schistosomiasis in Oyo. The diagnosis and subsequent control of this infection currently rely on expensive diagnostic tools that are laborious to use in the local endemic context, depend on well-trained personnel often lacking in rural communities, and are not field deployable. These factors limit the capacity for stakeholders within the health system in Oyo (Figure 1) to perform tasks targeting the diagnosis and subsequent control of schistosomiasis.

In investigating this issue, our findings revealed that new diagnostic devices that fit within the context of use will need to (1) be deployable to remote or distant communities, (2) be affordable, (3) identify and confirm infection status before treatment in patients with a diagnosis based on self-reporting, and (4) be optimized for the local setting and fit within local minimal infrastructural settings. Similarly, the study revealed that the context of use and application of potential new diagnostic devices is largely associated with the stakeholder tasks or diagnostic strategies employed. These findings will contribute to driving progress in developing new diagnostic devices for urinary schistosomiasis in Oyo and globally in several ways.^7^

The study findings will contribute to driving progress in developing new diagnostic devices for urinary schistosomiasis in Oyo and globally in several ways.

First, new diagnostic devices that are deployable will increase the capacity to diagnose and provide surveillance, especially in rural regions.^70^ Deployable diagnostic devices will support stakeholders such as DSNOs, NGOs, and researchers to perform pragmatic and supervisory tasks in schistosomiasis control and elimination. Such pragmatic and supervisory parts include gathering data, mapping, monitoring, and using information about schistosomiasis for program surveillance and planning, especially in various communities. Communities can receive schistosomiasis diagnostic health services irrespective of their location.^1^ However, deployable diagnostic devices will need to be sensitive for detecting low-level infections quickly; be safely transportable in cars, motorbikes, or bicycles; and have their own reliable power source because of the unstable power connectivity in rural regions.

Second, affordable diagnostic tests or devices that are supported by policies that reduce the overall cost of diagnosis will make diagnostic testing more affordable in Nigeria, where urinary schistosomiasis diagnostic tests costing between US$1 and US$2 (approximately ₦400 to ₦1000) are either lacking or not commercially available.^1^^,^^71^^–^^73^

Third, new diagnostic devices that can identify infection status before treatment will support the identification and prioritization of patients or areas in need of treatment. However, MDA programs have not led to morbidity reduction since estimates that prioritize the number of people eligible for treatment from the number of people treated has often been problematic.^74^^–^^77^ Overall, new diagnostic devices that can accurately identify infection status before treatment will support stakeholders at the health care, organizational, policy, and economic levels to prioritize MDAs in LGAs.

New diagnostic devices that can equip health facilities while requiring minimal infrastructure will increase diagnostic coverage especially in rural regions. In addition, equipping health facilities in rural regions will reduce the burden on patients who often travel long distances to access treatment and are unable to return for test results.^23^ Lastly, new diagnostic devices will need to support stakeholders in performing various tasks and strategies targeting urinary schistosomiasis within the context of Nigeria, as shown in this Q-methodological study. This support will ensure that new diagnostic devices are accepted and used by many end users in Oyo.^5^ Q-methodology served as a rigorous tool in this study because it is scientifically valid and reproducible in the domain of human-centered design. Human-centered design, or participatory design, seeks to understand user needs and insights to inform the design process.^78^ Design researchers can benefit from using Q-methodology as a participatory design tool to identify stakeholder needs and product-service design requirements, especially at the formative stage of the design-innovation process as in this study.

Q-methodology served as a rigorous tool in this study because it is scientifically valid and reproducible in the domain of human-centered design.

### Limitations

Although a small size of diverse respondents is not a limitation within this study, as large sample sizes are not required for a Q-methodology study, the findings are not generalizable beyond the small participant pool (Table 1). Furthermore, this study was conducted remotely using virtual digital platforms owing to COVID-19-related restrictions, which limited the ability to have in-person qualitative discussions. To ensure stakeholder viewpoints were extensively explored, written instructions and discussions within this study had to be clear and precise and solicited immediate feedback for clarifications. Considerable time was needed to prepare such concise instructions, which produced the key findings and their implications within this research. In-person conversations could have captured richer qualitative information and insights, which might have added additional explanatory value to this study.

## CONCLUSION

This study provides 2 key implications related to the development of potential new diagnostic devices and the stakeholders who perform tasks targeting urinary schistosomiasis control and elimination. First, medical device designers and technology companies should ensure the development of new diagnostic devices that can equip health care facilities with minimal infrastructure, be affordable, be deployable, and identify infection status before treatment. Second, the health care system and community stakeholders in Nigeria (Figure 1) should continuously participate and collaborate with the design industry in the human-centered design process of new diagnostic devices, thus ensuring stakeholder needs and viewpoints are considered in the design process. It is crucial to ensure that diagnostic devices are available at the point of need, are designed to function best within the local endemic health care context, and support stakeholders in performing tasks targeting control and eventual elimination of schistosomiasis ^5^^,^^20^^,^^25^^,^^30^ In conclusion, the findings from this study will guide the development of new diagnostic devices for schistosomiasis that match the contextual landscape and stakeholder diagnostic strategies in Oyo.

## Supplementary Material

GHSP-D-21-00780-supplement-6.xlsx

GHSP-D-21-00780-supplement-2.docx

GHSP-D-21-00780-supplement-5.xlsx

GHSP-D-21-00780-supplement-4.docx

GHSP-D-21-00780-supplement-1.docx

GHSP-D-21-00780-supplement-3.xlsx

## Notes

**Peer Reviewed**

**First published online:** August 4, 2022.
