# Supplementary material for: Stakeholders’ Perspectives on the Application of New Diagnostic Devices for Urinary Schistosomiasis in Oyo State, Nigeria: A Q-Methodology Approach
Source: Glob Health Sci Pract. 2022 Aug 30;10(4):e2100780. doi: 10.9745/GHSP-D-21-00780 (PMC9426976; doi:10.9745/GHSP-D-21-00780)
Supplement: GHSP-D-21-00780-supplement-2.docx [file GHSP-D-21-00780-supplement-2.docx]

**Supplement 2 Table 1. 40 selected statements with I-ICVI ratings and statement edits**

| **Cluster** | **No** | **Statement** | **I-CVI** | **Edited Statement** |
| --- | --- | --- | --- | --- |
| Context  (Use case) | 1 | Microscopy using a concentration technique is the recommended method to prove active schistosomiasis, despite its low sensitivity and need for expert users. | 0.88 | N/A |
|  | 2 | Diagnosis should include the identification of Schistosoma parasites in both humans and water sources that may be contaminated. | 0.75 | Diagnosis should include the identification of Schistosoma parasites in humans along with water sources that may be contaminated. |
|  | 3 | Mass screening and diagnosis should be carried out alongside mass drug administration with praziquantel. | 1.00 | N/A |
|  | 4 | Schistosomiasis surveillance enables programme managers to monitor the effectiveness of intervention strategies and identify which populations require continuing interventions. | 1.00 | N/A |
|  | 5 | The availability of Rapid Diagnostic Test (RDTs), which requires only minimal infrastructure, would improve diagnosis and surveillance simultaneously. | 1.00 | N/A |
|  | 6 | Implementing an affordable and simple point-of-care (POC) diagnostics solution will reduce the financial burden of equipment and personnel at each health facility. | 1.00 | N/A |
|  | 7 | Point-of-care diagnostics that can detect and confirm cases immediately will reduce the risk of missed or misdiagnosed cases. | 0.88 | N/A |
|  | 8 | The quantification of egg excretion helps to assess the transmission potential of populations living in endemic areas. | 1.00 | N/A |
|  | 9 | Schistosomiasis control programs should target school-aged children only. | 1.00 | N/A |
|  | 10 | Due to the low level of education and lack of training among community health workers, incorrect treatment is often prescribed. | 1.00 | N/A |
|  | 11 | Presenting data on the severity of schistosomiasis infection of specific locations will guide the development of strategies for effective case management and control elimination. | 1.00 | N/A |
|  | 12 | Passive case detection, based on people’s self-reporting, has been considered a less expensive strategy for the control of schistosomiasis. | 1.00 | N/A |
|  | 13 | Prevalence and intensity of infection is often higher among children than among adults. | 1.00 | N/A |
| Infrastructure and location | 14 | Schistosomiasis diagnosis should be done closest to the community as it reduces the time to carry samples back to the laboratory. | 1.00 | N/A |
|  | 15 | Diagnostic and treatment campaigns should target school-age children, adolescents and those whose occupations involve contact with infectious water (e.g fishing, farming, irrigation, and domestic tasks in water). | 1.00 | N/A |
|  | 16 | Simple, rapid point-of-care (POC) tests should be used in primary health care settings where patients often travel long distances to access healthcare facilities. | 1.00 | N/A |
|  | 17 | Diagnostic devices should be deployed in primary health care centres, clinics and health posts since they are the most lacking in equipment. | 1.00 | N/A |
|  | 18 | Testing of urine samples for schistosomiasis with school-based surveys should be done at the school location. | 1.00 | N/A |
|  | 19 | It is convenient to treat patients for schistosomiasis infection without a confirmed diagnosis due to the delay in receiving test results from referral hospitals. | 1.00 | N/A |
| Product  requirement | 20 | Schistosomiasis elimination calls for developing novel diagnostic tools with higher sensitivity and specificity than microscopes. | 0.88 | N/A |
|  | 21 | Diagnostic device for schistosomiasis with minimal to no sample preparation is ideal. | 0.88 | N/A |
|  | 22 | The diagnostic device should quantify eggs to provide an estimation of the number of people that have been exposed to schistosomiasis in a population. | 1.00 | The diagnostic device should quantify eggs to help estimation the number of people that have been exposed to schistosomiasis in a population. |
|  | 23 | Devices should be easy to use by medical personnel and health workers such as Community Health Extension Workers (CHEWS), Community Health Officers (CHO), Laboratory scientists to detect and diagnose schistosomiasis infected patients. | 1.00 | N/A |
|  | 24 | Patient samples should be processed in batches to get a faster turnaround time and increase the efficiency of sample processing during mass campaigns or sensitization meetings. | 1.00 | N/A |
|  | 25 | Ideal diagnostic approaches should allow the concurrent detection of several pathogens in different biological samples such as urine, blood and stool. | 1.00 | N/A |
|  | 26 | Diagnostic devices should be sensitive enough for detecting very light schistosomiasis infections. | 0.88 | N/A |
|  | 27 | Diagnostic devices should have their own reliable power sources due to the unstable power connectivity in rural and distant communities. | 0.88 | N/A |
|  | 28 | The best diagnostic devices should be easy to transport safely in cars, on motorbikes, and bicycles to remote locations. | 0.88 | N/A |
|  | 29 | Diagnostic devices should be compact and portable so that it can be easily deployed in the community. | 1.00 | N/A |
|  | 30 | Diagnostic devices/tests should identify and map out areas with a large spread of schistosomiasis and be able to trace the source of the disease. | 1.00 | N/A |
|  | 31 | Devices should be locally repaired and maintained by local technicians in case of breakdown. | 1.00 | N/A |
|  | 32 | The device should be easy to clean and disinfect to prevent re-contamination. | 1.00 | N/A |
| Roll-out  strategy | 33 | The cost per diagnostic test should be free (covered by the Government). | 1.00 | N/A |
|  | 34 | Cost per diagnostic test should be less than 1000 Naira (€2). | 1.00 | N/A |
|  | 35 | Mass drug administration campaigns should be accompanied by mass diagnostic and disease awareness campaigns. | 1.00 | N/A |
|  | 36 | Data from diagnostic devices should be accessible to stakeholders (local government, DSNO, MOH, Researchers and NGOs) to enhance planning. | 1.00 | N/A |
|  | 37 | New interventions should consider training the health care workers at the community level and the informal sector (PMVs and traditional medicine) to increase coverage to diagnostics. | 1.00 | N/A |
|  | 38 | Diagnostic tools for schistosomiasis should be deployed and used at the community level by PMVs and community mobilizers as they already serve as trusted stakeholders in the community. | 1.00 | N/A |
|  | 39 | The role of the village/community head is important in the acceptance of the new diagnostic device. | 1.00 | N/A |
|  | 40 | Patients with schistosomiasis should be tested before being treated. | 1.00 | N/A |

**Supplement 2 Table. Content validity and reliability ratings**

| **Table**. Content validity and reliability ratings for each of the 40 statements in the Q-set.  1 = not at all; 2 = somewhat, 3 = mostly, 4 = completely. | | | | |
| --- | --- | --- | --- | --- |
|  | **Not at all** | **Somewhat** | **Mostly** | **Completely** |
| The statement is clear and unambiguous as would be read by the participants | 1 | 2 | 3 | 4 |
| The statement illustrates heterogeneity (depth and breadth) | 1 | 2 | 3 | 4 |
