# Supplementary material for: Stakeholders’ Perspectives on the Application of New Diagnostic Devices for Urinary Schistosomiasis in Oyo State, Nigeria: A Q-Methodology Approach
Source: Glob Health Sci Pract. 2022 Aug 30;10(4):e2100780. doi: 10.9745/GHSP-D-21-00780 (PMC9426976; doi:10.9745/GHSP-D-21-00780)
Supplement: GHSP-D-21-00780-supplement-1.docx [file GHSP-D-21-00780-supplement-1.docx]

**Supplement 1: Collection of 150 statements from literature review and interviews**

**Research Questions -** What is the stakeholder perspective on the context of use and application of a new diagnostic device for urinary schistosomiasis in Oyo-State Nigeria?

# **Context *-*** *Context of use describes the interactions that occur between the following: stakeholders (actors), diagnostic device (object) and location.*

1. Reagent strips are therefore suggested as appropriate adjuncts for monitoring urogenital schistosomiasis control programmes.
2. Reagent strips for detecting macrohematuria in urine are useful tools for the indirect diagnosis of S. haematobium infection also in low-prevalence settings.
3. Diagnostic tests are central for adequate clinical patient management of Schistosoma-infected individuals and for successful control programmes aiming at schistosomiasis elimination.
4. Microscopy using concentrated technique is still the cornerstone to prove active schistosomiasis despite its low sensitivity and lack of laboratory experts.
5. The availability of Rapid Diagnostic Test, which requires only minimal infrastructure, such as Point of Care Circulating cathodic antigen cassette test (POC-CCA) urine cassette test would much improve diagnosis and at the same time surveillance in peripheral settings.
6. Surveillance is the cornerstone of successful disease control and elimination programmes, as it enables programme managers to monitor the effectiveness of intervention strategies and identify which populations require continuing interventions.
7. The role of diagnostics in the control of selected infectious diseases in developing countries suggest Mass treatment as a control strategy.
8. Microscopy for diagnostic tests of stool and urine is positive on the role of diagnostic tests & Case-management but negative on the screening and Monitor impact of control programmes for surveillance.
9. The quantification of egg excretion also helps to assess the transmission potential of populations living in endemic areas and determine the efficacy of praziquantel treatment in terms of egg reduction rates.
10. Schistosomiasis elimination calls for developing novel diagnostic tools with a high sensitivity and high specificity.
11. Drive towards test-and-treat strategy in close-to-elimination settings or for post-elimination surveillance might be more cost-effective, less drug- and resource-wasting, and ethically more appropriate than the large-scale distribution of drugs to mostly uninfected people.
12. Schistosomiasis control programs should target school-aged children and give praziquantel for the treatment of schistosomiasis.
13. Schistosomiasis control and elimination should be approached by large-scale deworming exercise of school-age children in endemic zones with praziquantel.
14. Diagnosis among adults should be covered by mass administration of praziquantel.
15. Programmatic parts of schistosomiasis control as well as gathering and using information about schistosomiasis for program planning.
16. People’s own knowledge of signs and symptoms as recorded in questionnaires has proved a valid and very cost-effective way of identifying and monitoring hot spots of transmission at community level.
17. Community-directed treatment in which community members organize and effectuate the distribution of medication for deworming after collecting the drugs from central stocks is a new approach to morbidity control.
18. In countries without schistosomiasis control programmes, equipping healthcare facilities for passive case detection is the only option.
19. The control and elimination programmes are the principal strategy for control of schistosomiasis.
20. The case management of individual cases at the primary health care level.
21. The control and elimination program provides annual mass treatment of praziquantel for school-age children aged 5 to 14, who are known as the most heavily infected part of the population.
22. Infected individuals do not visit a formal health facility when they have symptoms. Due to low awareness of schistosomiasis amongst community members, bloody urine is not perceived as a symptom of a medical condition.
23. The education level of community health workers is low, and they assume a low prevalence of schistosomiasis.
24. As a result, wrong treatment is prescribed, and light and asymptomatic cases are never recognized.
25. WHO states that cases of urinary schistosomiasis should only be suspected when an infected individual has bloody urine? Schistosomiasis symptoms are often misinterpreted for other conditions, like STIs or malaria.
26. Epidemiological surveys through which cases are confirmed, depend largely on data from children alone, so the current true disease prevalence is unknown.
27. Dipsticks and questionnaires are available to field-deployable methods, but they lack sensitivity.
28. Availability of diagnostics and disease detection rates by the health worker in the community were challenging factors.
29. Even when the case is suspected, the health care providers face challenges in providing laboratory confirmation of the case due to unavailability of the diagnostic equipment and personnel.
30. Active involvement of the community members for sensitization and health education will improve the general awareness of schistosomiasis by overcoming mis-associations around the symptoms and the passive attitudes in health-seeking behaviours.
31. Presenting data specific to their local context will provide the health workers with awareness of the severity and urgency and consequently improve accommodation by providing the appropriate care for schistosomiasis. The prevalence study will generate needed evidence and guide the development of appropriate strategies for effective implementation of case management.
32. Implementing an affordable and simple point-of-care diagnostics solution will reduce the financial burden of equipment and personnel at each health facility. Point-of-diagnostics can confirm the detected cases immediately and will reduce the risk of missed or misdiagnosed cases.
33. Serological detection of anti-schistosome antibodies in human blood is commonly employed to determine whether or not an individual has been previously exposed to schistosomes.
34. DNA detection tests are hardly used for clinical diagnosis within Schistosoma- endemic countries because they require expensive laboratory equipment and highly skilled personnel.
35. Antigen detection tests. Novel tools showing a very high diagnostic accuracy
36. For the diagnosis of early-stage infections, there is a need for ultra-sensitive blood-based diagnostic tests, which can detect Schistosoma infection even before serology and microscopy become positive.
37. Diagnosis of Schistosomiasis should identify and map out areas with large spread of schistosomiasis and trace the source of the disease.
38. Diagnosis includes both identification of Schistosoma parasites in both humans and water sources that are contaminated.
39. Massive screening should be carried out before drug mass drug administration with praziquantel. This will help identify high risk areas.
40. New devices for Schistosomiasis infection mapping should be done closest to the community. It reduces the time to carry samples back to the laboratory.
41. We also heard that the presence of blood in the urine can be confusing because malaria and STI can have an orange-coloured urine.
42. I think for the adults, this should be the first step. They have to be diagnosed before they can be given the drugs. 20 years and above, they should be tested to know whether they have the disease or not.
43. I do not know the rate at which the adults are infected with schistosomiasis. If we had high endemic levels of schistosomiasis with the prevalence above 50, they will definitely be included but currently, the prevalence is moderate and below 50.
44. Some end up going to the farm and they stay long on the farm before they return from the farm. It's nightfall and then, but we need to travel back to our facility. - Lab tech.
    1. New devices for Schistosomiasis infection mapping should be done closest to the community.
45. Ah, well – we feel that, it’s {eh} we have gone there, we have collected, you know, a sample we have given out the test and the only thing we need is to come back to our facility to start working on the result. Most times we are after the data that we collect. We don’t go back for monitoring or evaluation.
46. For instance, we use RDTs to diagnose malaria and you can make the diagnosis while seeing your patient instead of sending them to the lab.

# **Infrastructure.** *In what infrastructure and location should the new diagnostic device be deployed to make its first use? For example, should the new device be deployed in a laboratory setting in a primary health centre, health post, or in teaching/research facilities, field-deployable stations, etc.*

1. Self-reported blood in urine is a rapid and low-cost diagnostic technique for urinary schistosomiasis.
2. Simple School base questionnaires is an effective rapid and low-cost diagnostic technique to assess and diagnose urinary schistosomiasis infection in high affected communities.
3. Schistosomiasis diagnostic centres should be areas water resource development areas - e.g., fishing or swamp communities.
4. Simple, rapid POINT-OF-CARE (POC) test should be used in primary health care settings where there may be no electricity for equipment or refrigerators (for the storage of reagents), and where patients often travel long distances and may therefore be unable to return for test results.
5. Schistosomiasis detection should be available in national reference laboratories allowing their use not only for research purposes, but also in clinical practice as a diagnostic tool to guide individual patient management.
6. Standard laboratories remain the cornerstone to prove active schistosomiasis.
7. Schistosomiasis diagnostics should be cantered in district and rural dispensaries where no or only limited diagnostic laboratory infrastructure is available.
8. WHO identifies school-age children, adolescents and those whose occupations involve contact with infectious water (fishermen, farmers, irrigation workers, women doing domestic tasks) as high-risk groups that should be the main target group for treatment campaigns?
9. Diagnostics should be targeted towards children. Prevalence and intensity of infection is often higher among children than among adults (and thus children account for a larger part of the contamination) and reinfection rates are also often higher in children than in adults.
10. Teachers’ role in administering questionnaires for community diagnosis should be emphasized as a form of community participation involving non-specialists.
11. Passive case detection, based on people’s self-reporting, has been considered a less expensive strategy based on the integration of schistosomiasis control into the regular health services.
12. Case finding and management of schistosomiasis at primary health care level, particularly in areas where S. haematobium and S.mansoni coexist, should be improved.
13. The DSNO is responsible for getting a sample tested to confirm the case. This often results in extra work, since she or he has to pick up the sample at a PHC facility and bring it to an approved laboratory.
14. Not all suspected cases are reported, and the DSNO does not always have time to get the case confirmed.
15. Hospitals are inaccessible due to long distances, poor roads quality and expensive transport fares.
16. A community health worker does not see the patient after referral to a hospital or administering treatment. There is no check-up or test to confirm the cure of the patient.
17. Community health workers do not know if the patient went to the hospital and if the patient has recovered.
18. Microscopes are fragile and expensive, and filters for sample preparation may not be readily available in Nigeria. As a result, testing for the school-based survey was performed in hospital laboratories instead of at location of sample collection.
19. School-based surveys should be performed at the location of sample collection instead at the hospital laboratories.
20. Community health workers should use the diagnostic device in the community that requires simple sample preparation and device interactions.
21. Referral to another diagnostic facility is possible but the delay in receiving the result led to treating the patients without confirmation for the sake of convenience.
22. Lastly, the referral to other facilities brings more burden of time and costs for the patients, which relates to the issues of accessibility and affordability.
23. There is a need for a prevalence study focusing on other high-risk groups including the adults who frequently interact with water.
24. New interventions should consider training the health care providers at the community level and the informal sector (PMVs and traditional medicine) to enhance collaboration between them. This will improve the awareness in the community.
25. Diagnostic devices should be deployed in primary health care centers, clinics, health posts since they are the most lacking in equipment and closest to the community for patients to seek care.
    1. For instance, type 1 which is the health post must have at least two rooms, type 2 must have a minimum of 5 rooms, type 3 should have a minimum of 14 rooms to be an official primary health care center. It must have a laboratory section, a pharmacy section and all the other compliments of health services. Under type 3, it must have these sections to be a proper health care center.
26. Diagnostic device should be deployed in marginalised areas like slums were the rate of human interaction is high and access to healthcare is low.
27. Health facilities closest to the people lie wards (Local government areas) are in massive need of schistosomiasis diagnostic tools.
    1. There is no machine. We do have labs but we are limited to some tests to be carried out at the LGA level
28. Diagnostic tools for Schistosomiasis should be deployed and accessed at PMV as they already serve as medicine supply stores in the community.
    1. They prefer to go to patent medicine vendors to just get one or two drugs that are irrelevant to their health problems.
29. Diagnostic devices should be deployed where CHEWS are stationed to work. There are the closest to the community and should be able to diagnose cases.
30. you should target the people in the slummy settlements, rural settlements, populations around the river bank areas. These populations are more exposed because their children go into the rivers to bathe. Some people can even come from nearby communities, can go into the rivers to bathe, adults with no prior knowledge to the disease are also at risk.
31. So like the Idikan health centre, I do go there to run some basic tests on some patients {you know} after the, the clinicians or doctors {you know} when they see the patient and, of course, you know when you want to run some tests. So, when I got there, I felt that it would be very wise for us to have a close-by point of care laboratory unit.
32. Yes, they do. Most of them have my phone number. They do call me to tell me they are having a headache and so on.

**Product Requirement:** *What product requirements are needed in the new diagnostic device? Aspects of product requirements involve: product performance, cost, cleaning, power connectivity., etc.*

1. Diagnostics for schistosomiasis should not depend on a high skilled technologist.
2. Medical personnel such as CHEWS/CHO/DSNO/ should be able to diagnose Schistosomiasis.
3. Rapid identification of schistosomiasis in high-risk communities should be instantaneous.
4. Immediate and instantaneous results on diagnostics of schistosomiasis should be applied in communities.
5. Diagnostic test for schistosomiasis would be widely available in remote areas
6. One trained technician can manage several dozen such as UCP-LF CAA or PCR assays per day to allow the use in large screening programmes for near-elimination surveillance.
7. Diagnostic test results should be available in 3 - 4 hours.
8. Tests should be processed in batches.
9. Simple, rapid POINT-OF-CARE (POC) TESTS that can be used to guide treatment as they are simple to perform, produce rapid visual readouts and often require no equipment.
10. A diagnostics test should be ASSURED - A = affordable by the affected individuals; S = sensitive; S = specific; U = user-friendly; R = rapid turn-around time and robust performance (e.g., reagents tolerate tropical climate); E = equipment-free; and D = delivered to those in need.
11. Diagnostic test should be below $1 per test - How much is the current test?
12. Ideal diagnostic approaches would allow the concurrent detection of several pathogens in one biological sample (e.g., urine or stool).
13. Schistosomiasis control and elimination programmes to adopt the most sensitive (for detection of very light infection intensities).
14. highly specific tests (to minimise the number of false-positive samples in areas approaching elimination of schistosomiasis).
15. non-invasive test procedure, the short turn-around time (results available within less than 30 min) and the high sensitivity of the urine-based POC-CCA are needed for the diagnostics.
16. there is a need for more sensitive, cheaper, and easy to use devices for the diagnosis and control of schistosomiasis.
17. device improving the diagnostic process and increasing efficiency, especially in hard-to-reach areas.
18. At community level, women’s access to treatment may be hampered by lack of access to cash and possible restrictions on their movements hindering visits to health facilities for health problems that are not considered life-threatening.
19. Also, the manual microscopic examination of the filtered urine sample is time consuming and prone to human error.
20. It is difficult for infected individuals in rural communities to get diagnosed, since there is a limited number of PHC facilities. The health posts and clinics that are available are understaffed and lack resources, so they cannot carry out community-based services.
21. Microscopes are fragile and expensive, and filters for sample preparation may not be readily available in Nigeria.
22. Power requirements (minimal 8-hour operation between charges) and connectivity (via mobile network) are ideal.
23. The device should produce same day results so that the infected individuals can get treatment(s) right after the confirmation.
24. The device should be transported from one location to another, the test kit should be able to tolerate transportation stress.
25. Charging and calibration should be not required during the day to minimize the time.
26. The device has a throughput of at least five samples per hour, and the results should be available before the end of the sensitization meeting.
27. Ideally, the device can save the location data and number of infected samples to identify the location of infected water bodies.
28. The availability of smart diagnostics will be beneficial to detect light infections or asymptomatic cases and avoid misdiagnosis.
29. First, I think it is the compact, portable and deployed in the community.
30. Device should, store, map-location, take images of water sources and transfer data instantly.
31. I think it makes it really easy if you really do not need so much skills like having to manipulate, read and diagnose Schistosomiasis.
32. It is good to quantify eggs because it gives an idea of the extent the people have been exposed and what one should expect in terms of treatments or duration and so on.
33. Patient diagnostic should be free or below 20cents (100 Naira)
34. Cost of device should range from being free for PH centres to between 10000 - 50000 Naira bigger hospitals or researchers.
35. You don't need so much expect training to be able to use it.
36. Something that it's not solely dependent on electricity, that’s our problem here, except you don't want to face reality.
37. something that results can be ready in the same day, not go back and come back in 3 days.
38. Electricity is something we lack. You also have to factor rural environment where there is no electricity at all.
39. If you want to do microscopy, it should be about 1000 naira. It actually should not be more than 1000 naira. This is because PHC is basically next to taking care of people who are really poor.
40. Power supply is also important because if you bring the device and there is no electricity to power it, it will be another issue.
41. Most of the time, there is no power supply to carry out microscopy.
42. We want to process it the sample and then we say if this person is Schisto-positive or negative and how many eggs in the sample are something like that. So, first thing I can think of is that it should be cheap, it shouldn’t be too expensive because now people already have struggle going to the lab.
43. Yea, they pay 300 naira for RDT I know before is it good price the price 300 naira.
44. yeah, maybe power outage between the time the{erm} light goes on and generator comes on. Those are the limitations
45. Should it be easier to repair?
46. Then I think we planned to go to Kwara state, which we planned to go with our equipment, along the line, I think the - portable and deployable on the go
47. Maybe on the average between 5 and 10 urinalyses on the device?
48. At least the sensitivity should be the first thing that you prioritise so that even if there is maybe one in hundred that thing should be able to detect it.
49. Currently we no power supply in the hospital. We have an old generator that runs the diagnostic devices
50. Health workers sometimes forget to report cases. For example, the DSNO did report a case because they did not have time.
51. The waiting time between sending samples to the lab and getting results from the lab is reduced.
52. We are members of the Cele Church. It is our tradition to bathe in the streams.
53. The road is really bad especially during the rainy season and transport is also expensive. The cost of treatment always put some people off. Most times, they cannot afford to purchase the drugs prescribed to them.
54. The government should provide machines to ensure that the tests are faster for us. Although, the faster the tests, the more expensive they will be and poverty is still a major problem in this community.
55. They will willingly pay anything that costs two hundred naira. If it costs more than that, they will begin to dread it. They will say that they have not even had anything to eat. Poverty is a serious problem in this community.

# **ROLE-OUT STRATEGY:** *What strategies should be employed to guarantee a successful rollout of the new diagnostic device?*

1. More active versions of control at primary health care level have combined treatment campaigns with improvements of water supply and sanitation and other control efforts in endemic areas. It has been argued that the starting point of such interventions should be based on local concerns and priorities.
2. Control programmes should explore how they can best work together with other programmes and initiatives, such as the healthy child initiative, health promoting schools, etc
3. Poor understanding of the disease also leads to reduced patient compliance to free treatment and risk-taking behaviour (e.g., exposure to endemic freshwater). If a patient knows about the disease and knows that they are infected (e.g., confirmed by diagnosis) they are more likely to comply with treatment on an annual basis.
4. The confirmed case should be shared with the DSNO in accordance with the guidelines.
5. The device should include instructions on treatment and health education.
6. Participation in disease mapping for schistosomiasis has to be free for the community members.
7. Before the results are available, health education can take place to increase awareness.
8. The disease awareness was a major barrier for the patients at the beginning of the health-seeking pathway, followed by accessibility and affordability.
   1. Lack of disease awareness was one of the main barriers.
   2. I think the awareness has helped a lot and that is why we have not seen cases. There is nothing to be hidden.
9. Data should be retrievable. For planning - been reported by any facility, it is our duty to extract the information and put it under our Local Government information and then send that information to the state, WHO and national authorities if necessary.
10. The health workers there will treat the patient and document it. We will then send the record to the state.
11. Do you think the awareness changed after the exercise?
12. Children are more expressive while the parents are not completely open about how they feel. They feel more shame than children. The children find it easier to open up. Some pregnant women would rather give birth in their huts. And this is very common in Yoruba land.
13. The first thing is that a training programme about schistosomiasis should be introduced. The staff need to be educated and there should be awareness in the community so that people will know what schistosomiasis is, the causes and what they should do if they see any of the signs.
14. I know that they pay 800 naira for the extensive tests in the local government.
15. If I do not go out to sensitize people, they will not come out to report to me. We also have some people in the community that we call community informants and we train them often so that whenever they set their eyes on something of such nature, they can immediately notify me and I can go to make investigations.
16. we have social mobilization committee where we meet with them and teach them the necessary things.
17. Diagnostic device for schistosomiasis with minimal to no sample preparation is ideal.

**Stakeholder interview list.**

| **Healthcare Level** | **Stakeholder** | **Interview count** |
| --- | --- | --- |
| Policy | NGO | 1 |
|  | Academia/Researcher | 2 |
|  |  |  |
| Organizational | Primary Healthcare  Coordinator (PHC) | 1 |
|  | Medical Officer of Health (MOH) | 1 |
|  | Disease Surveillance Notification Officer (DSNO) | 2 |
|  | Neglected Tropical Disease Officer (NTD) | 3 |
|  | Teacher | 6 |
|  |  |  |
| Healthcare | Doctors | 1 |
|  | Health Worker | 4 |
|  | Lab Technician | 4 |
|  | Community Health Worker (CHO) | 2 |
|  |  |  |
| Community | Patient/Guardian | 5 |
|  | Community Mobilizer | 1 |
|  | Traditional Healer | 1 |
|  | Community Leader | 1 |
|  |  |  |

**References (Literature review)**

1. Adekeye O, Dean L, Dixon R. Community Engagement in Neglected Tropical Disease Treatment in Nigeria: Rethinking the Needs of Varying Contexts. (2017). Available online at: <https://countdown.lstmed.ac.uk/sites/default/files/content/centre_page/attachments/CommunityEngagement_final.pdf> (accessed April 28, 2020).
2. Adenowo AF, Oyinloye BE, Ogunyinka BI, Kappo AP. Impact of human schistosomiasis in sub-Saharan Africa. Braz J Infect Dis. (2015) 19:196–205. doi: 10.1016/j.bjid.2014.11.004
3. Agbana, T., et al. Schistoscope: Towards a locally producible smart diagnostic device for Schistosomiasis in Nigeria. in 2019 IEEE Global Humanitarian Technology Conference (GHTC). 2019
4. Agbana, T.E., et al., Imaging & identification of malaria parasites using cellphone . microscope with a ball lens. PloS one, 2018.13(10): p. e0205020-e0205020.
5. Agbana T, Nijman P, Hoeber M, Grootheest D van, Diepen A van, Lieshout L van, et al. Detection of Schistosoma haematobium using lensless imaging and flow cytometry, a proof of principle study. In: Optical Diagnostics and Sensing XX: Toward Point-of-Care Diagnostics. San Francisco, CA (2020). doi: 10.1117/12.2545220
6. Ajibola O, Gulumbe B, Eze A, Obishakin E. Tools for detection of schistosomiasis in resource limited settings. Med Sci. (2018) 6:39.doi: 10.3390/medsci6020039
7. Akanbi MO, Ocheke AN, Agaba PA, Daniyam CA, Agaba EI, Okeke EN, et al. Use of electronic health records in sub-Saha- ran Africa: progress and challenges. Journal of Medicine in the Tropics 2012;14(1):1–6. PMid:25243111; PMCid:PMC4167769
8. Bassiouny HK, Hasab AA, El-Nimr NA, Al-Shibani LA, Al- Waleedi AA. Rapid diagnosis of schistosomiasis in Yemen using a simple questionnaire and urine reagent strips. East Mediterr Health J2014;20:242–9.
9. Beran D, Lazo-Porras M, Cardenas MK, Chappuis F, Damasceno A, Jha N, et al. Moving from formative research to co-creation of interventions: insights from a community health system project in Mozambique, Nepal and Peru. BMJ Glob Health. (2018) 3:e001183. doi: 10.1136/bmjgh-2018-001183
10. Braun-Munzinger RA, Southgate BA. Repeatability and reproducibility of egg counts of Schistosoma haematobium in urine. Trop Med Parasitol.(1992) 43:149–54.
11. Bruun, B., & Aagaard-Hansen, J. (2008). The social context of schistosomiasis and its control: an introduction and annotated bibliography. World Health Organization.
12. Centers for Disease Control and Prevention. Schistosomiasis. Centers for Disease Control and Prevention (2019). Available online at: <https://www.cdc>. gov/dpdx/schistosomiasis/index.html (accessed May 20, 2020).
13. Chitsulo L, Engels D, Montresor A, Savioli L. The global status of schistosomiasis and its control. Acta Trop. (2000) 77:41–51. doi: 10.1016/S0001-706X(00)00122-4
14. Colley DG, Binder S, Campbell C, King CH, Tchuem Tchuenté LA, N’Goran EK, et al. A five-country evaluation of a point-of-care circulating cathodic antigen urine assay for the prevalence of Schistosoma mansoni. Am J Trop Med Hyg 2013;88:426–32.
15. Corstjens PLAM, de Dood CJ, Kornelis D, Fat EM, Wilson RA, Kariuki TM, et al. Tools for diagnosis, monitoring and screening of Schistosoma infections utilizing lateral-flow based assays and upcon- verting phosphor labels. Parasitology 2014;141:1841 – 55.
16. Corstjens PLAM, van Lieshout L, Zuiderwijk M, Kornelis D, Tanke HJ, Deelder AM, et al. Up-converting phosphor technology-based lateral flow assay for detection of Schistosoma circulating anodic antigen inserum. J Clin Microbiol 2008;46:171 – 6.
17. Coulibaly JT, Knopp S, N’Guessan NA, Silué KD, Fürst T, Lohourignon LK, et al. Accuracy of urine circulating cathodic antigen (CCA) test for Schistosoma mansoni diagnosis in different settings of Côte d’Ivoire. PLoS Negl Trop Dis 2011;5:e1384.
18. Coulibaly JT, N’Gbesso YK, Knopp S, N’Guessan NA, Silué KD, van Dam GJ, et al. Accuracy of urine circulating cathodic antigen test for the diagnosis of Schistosoma mansoni in preschool-aged children before and after treatment. PLoS Negl Trop Dis 2013;7:e2109.
19. Dawaki, S.; Al-Mekhlafi, H.M.; Ithoi, I.; Ibrahim, J.; Abdulsalam, A.M.; Ahmed, A.; Sady, H.; Nasr, N.A.; Atroosh, W.M. The menace of schistosomiasis in Nigeria: Knowledge, attitude, and practices regarding schistosomiasis among rural communities in Kano State. PLoS ONE 2015, 10, e0143667. [CrossRef]
20. Diehl JC, Oyibo P, Agbana T, Jujjavarapu S, Van G-Y, OyiboW. Schistoscope: smartphone vs. raspberry Pi based low cost diagnostic device for urinary schistosomiasis. In: 10th IEEE Global Humanitarian Technology Conference (GHTC). Seattle,WA: IEEE (2020). p. 1–8.
21. El Ridi RAF, Tallima HA-M. Novel therapeutic and prevention approaches for schistosomiasis: review. J Adv Res. (2013) 4:467–78. doi: 10.1016/j.jare.2012.05.002
22. Emukah E, Gutman J, Eguagie J, Miri ES, Yinkore P, Okocha N, et al. Urine heme dipsticks are useful in monitoring the impact of praziquantel treatment on Schistosoma haematobium in sentinel communities of Delta state, Nigeria. Acta Trop 2012;122:126–31.
23. Emukah E, Gutman J, Eguagie J, Miri ES, Yinkore P, Okocha N, et al. Urine heme dipsticks are useful in monitoring the impact of praziquantel treatment on Schistosoma haematobium in sentinel communities of Delta state, Nigeria. Acta Trop 2012;122:126–31.
24. Ezeh, C.O., et al., Urinary schistosomiasis in Nigeria: a 50 year review of prevalence, distribution and disease burden. Parasite (Paris, France), 2019. 26: p. 19-19.
25. Federal Ministry of Health. NIGERIA NTD Master Plan: 2015-2020. Federal Ministry of Health (2015). Available online at: <http://espen.afro.who.int/> system/files/content/resources/NIGERIA_NTD_Master_Plan_2015_2020. pdf (accessed April 20, 2020).
26. Glinz D, Silué KD, Knopp S, Lohourignon LK, Yao KP, Steinmann P, et al. Comparing diagnostic accuracy of Kato–Katz, Koga agar plate, ether-concentration, and FLOTAC for Schistosoma mansoni and soiltransmitted helminths. PLoS Negl Trop Dis 2010;4:e754.
27. Gryseels B, Polman K, Clerinx J, Kestens L. Human schistosomiasis. Lancet 2006;368:1106–18.
28. Hoekstra PT, Casacuberta Partal M, Amoah AS, van Lieshout L, Corstjens PLAM, Tsonaka S, et al. Repeated doses of praziquantel in schistosomiasis treatment (RePST) – single versus multiple praziquantel treatments in school-aged children in Côte d’Ivoire: a study protocol for an open-label, randomised controlled trial. BMC Infect Dis. (2018) 18:662. doi: 10.1186/s12879-018-3554-2
29. ​Holmström, O., et al., Point-of-care mobile digital microscopy and deep learning for the detection of soil-transmitted helminths and Schistosoma haematobium. Global health action, 2017. 10(sup3): p. 1337325-1337325.
30. ​Hopkins, D.R., et al., Lymphatic filariasis elimination and schistosomiasis control in combination with onchocerciasis control in Nigeria. The American journal of tropical medicine and hygiene, 2002. 67(3): p. 266-272.
31. Hotez PJ, Alvarado M, Basáñez MG, Bolliger I, Bourne R, Boussinesq M, et al. The global burden of disease study 2010: interpretation and implications for the neglected tropical diseases. PLoS Negl Trop Dis 2014;8:e2865.
32. Hotez PJ, Fenwick A, Savioli L, Molyneux DH. Rescuing the bottom billion through control of neglected tropical diseases. Lancet. (2009) 373:1570–5. doi: 10.1016/S0140-6736(09)60233-6
33. Hotez PJ, Kamath A. Neglected tropical diseases in Sub-Saharan Africa: review of their prevalence, distribution, and disease burden. PLoS Negl Trop Dis. (2009) 3:e412. doi: 10.1371/journal.pntd.0000412
34. Isere E, Fatiregun A, Ajayi I. An overview of disease surveillance and notification system in Nigeria and the roles of clinicians in disease outbreak prevention and control. Niger Med J. (2015) 56:161–8. doi: 10.4103/0300-1652.160347
35. J. Utzinger, S. L. Becker, L. van Lieshout, G. J. van Dam and S. Knopp. New diagnostic tools in schistosomiasis. Clinical Microbiology and Infection © 2015 European Society of Clinical Microbiology and Infectious Diseases. Published by Elsevier Ltd. All rights reserved, CMI, 21, 529–542
36. Jane B. Paige and Karen H. Morin Study: Q-Sample Construction: A Critical Step for a Q-Methodological. West J Nurs Res published online 4 August 2014. DOI: 10.1177/0193945914545177.
37. King CH, Bertsch D. Meta-analysis of urine heme dipstick diagnosis of Schistosoma haematobium infection, including low prevalence and previously-treated populations. PLoS Negl Trop Dis 2013;7:e2431.
38. Knopp S, Becker SL, Ingram K, Keiser J, Utzinger J. Diagnosis and treatment of schistosomiasis in children in the era of intensified control. Expert Rev Anti Infect Ther 2013;11:1237–58.
39. Knopp S, Corstjens PLAM, Koukounari A, Cercamondi CI, Ame SM, de Dood CJ, et al. Sensitivity and specificity of a urine circulating anodic antigen test for the diagnosis of Schistosoma haematobium in low endemic settings. PLoS Negl Trop Dis 2015;9:e0003752.
40. Knopp S, Person B, Ame SM, Mohammed KA, Ali SM, Khamis IS, et al. Elimination of schistosomiasis transmission in Zanzibar: baseline findings before the onset of a randomized intervention trial. PLoS Negl Trop Dis 2013;7:e2474.
41. Knopp S, Salim N, Schindler T, Karagiannis Voules DA, Rothen J, Lweno O, et al. Diagnostic accuracy of Kato-Katz, FLOTAC, Baermann and PCR methods for the detection of light intensity hookworm and Strongyloides stercoralis infections in Tanzania. Am J Trop Med 2014;90:535–45.
42. Knopp S, Salim N, Schindler T, Karagiannis Voules DA, Rothen J, Lweno O, et al. Diagnostic accuracy of Kato-Katz, FLOTAC, Baermann and PCR methods for the detection of light intensity hookworm and Strongyloides stercoralis infections in Tanzania. Am J Trop Med 2014;90:535–45.
43. Le L, Hsieh MH. Diagnosing urogenital schistosomiasis: dealing with diminishing returns. Trends Parasitol. (2017) 33:378–87. doi: 10.1016/j.pt.2016.12.009
44. Lengeler C, Utzinger J, Tanner M. Questionnaires for rapid screening of schistosomiasis in sub-Saharan Africa. Bull World Health Organ 2002;80:235–42.
45. LoVerde PT. Schistosomiasis. In: Toledo R, Bernard F, editors. Digenetic rematodes. 2nd ed. New York, NY: Springer-Verlag (2019). p. 45–70.
46. [Mabey D](https://docs.google.com/document/d/1OuODgPGH3bLqyIAdcXAlcPH1jYGb_q3m4gTNmaTFb4E/edit#bookmark=id.73nlwbhrgpc4), Peeling RW, Ustianowski A, Perkins MD. Diagnostics for the developing world. Nat Rev Microbiol 2004;2:231–40.
47. Mafe, M.A., et al., Effectiveness of different approaches to mass delivery of praziquantel among school-aged children in rural communities in Nigeria. Acta Tropica, 2005. 93(2): p. 181-190.
48. Montresor A, Crompton DWT, Hall A, Bundy DAP, Savioli L. Guidelines for the evaluation of soil-transmitted helminthiasis and schistosomiasis at community level. Geneva: World Health Organization; 1998. p. 1–49.
49. Mostert-Phipps N, Pottas D and Korpela M. Guidelines to encourage the adoption and meaningful use of health information technologies in the South African healthcare landscape. Studies in Health Technology and Informatics 2013;192:147–51.
50. Munisi DZ, Buza J, Mpolya EA, Angelo T, Kinung’hi SM. The efficacy of single-dose versus double-dose praziquantel treatments on schistosoma mansoni infections: its implication on undernutrition and anaemia among primary schoolchildren in two on-shore communities, Northwestern Tanzania. BioMed Res Int. (2017) 2017:7035025. doi: 10.1155/2017/7035025
51. Murray CJL, Vos T, Lozano R, Naghavi M, Flaxman AD, Michaud C, et al. Disability-adjusted life years (DALYs) for 291 diseases and injuries in 21 regions, 1990-2010: a systematic analysis for the Global Burden of Disease Study 2010. Lancet 2012;380:2197–223.
52. Mwinzi PNM, Kittur N, Ochola E, Cooper PJ, Campbell CH, King CH, et al. Additional evaluation of the point-of-contact circulating cathodic antigen assay for Schistosoma mansoni infection. Frontiers Public Health 2015;3.
53. Mwinzi PNM, Kittur N, Ochola E, Cooper PJ, Campbell CH, King CH, et al. Additional evaluation of the point-of-contact circulating cathodic antigen assay for Schistosoma mansoni infection. Frontiers Public Health 2015;3.
54. Nebe OJ, Anagbogu IN, Ngige EN, Isiyaku S, Adamani WEM A, Nwobi BC. Epidemiological mapping of schistosomiasis and soil transmitted helminthiasis in 19 states and the federal capital territory (fct), Nigeria. Am J Trop Med Hyg. (2017) 95(Suppl. 5):559. doi: 10.4269/ajtmh.abstract2016
55. Nigeria, F.M.o.H., Report on Epidemiological Mapping of Schistosomiasis and Soil Transmitted Helminthiasis in 19 States and the FCT. 2015, Nigeria.
56. Olaseha IO, Sridhar MKC. Participatory action research: community diagnosis and intervention in controlling urinary schistosomiasis in an urban community in Ibadan, Nigeria. Int Q Community Health Educ. (2005) 24:153–60. doi: 10.2190/CBYM-94N2-E7DH-QRAL
57. Onasanya A, Keshinro M, Oladepo O, Van Engelen J, Diehl JC. A Stakeholder Analysis of Schistosomiasis Diagnostic Landscape in South-West Nigeria: Insights for Diagnostics Co-creation. Front Public Health. 2020;8:564381. Published 2020 Oct 30. doi:10.3389/fpubh.2020.564381
58. Onwuegbuzie AJ and Frels RK. Using Q methodology in the literature review process: a mixed research approach. Journal of Educational Issues 2015;1(2):90–109. Available from: https:// doi.org/10.5296/jei.v1i2.8396.
59. Peters PA, Mahmoud AA, Warren KS, Ouma JH, Siongok TK. Field studies of a rapid, accurate means of quantifying Schistosoma haema- tobium eggs in urine samples. Bull World Health Organ 1976;54:159 – 62
60. Ramlo S. Mixed method lessons learned from 80 years of Q methodology. Journal of Mixed Methods Research 2016;10(1):28‒45.
61. [Rollinson D, Knopp S, Levitz S, Stothard JR, Tchuem Tchuenté LA, Garba A, et al. Time to set the agenda for schistosomiasis elimination. Acta Trop 2013;128:423–40.](https://docs.google.com/document/d/1OuODgPGH3bLqyIAdcXAlcPH1jYGb_q3m4gTNmaTFb4E/edit#bookmark=id.ddvn8yw700uj)
62. Ross AG, Chau TN, Inobaya MT, Olveda RM, Li Y, Harn DA. A new global strategy for the elimination of schistosomiasis. Int J Infect Dis. 2017 Jan;54:130-137. doi: 10.1016/j.ijid.2016.09.023. Epub 2016 Dec 12. PMID: 27939558 sciences.
63. Shane HL, Verani JR, Abudho B, Montgomery SP, Blackstock AJ, Mwinzi PN, et al. Evaluation of urine CCA assays for detection of Schistosoma mansoni infection in Western Kenya. PLoS Negl Trop Dis 2011;5:e951.
64. Shane HL, Verani JR, Abudho B, Montgomery SP, Blackstock AJ, Mwinzi PN, et al. Evaluation of urine CCA assays for detection of Schistosoma mansoni infection in Western Kenya. PLoS Negl Trop Dis 2011;5:e951.
65. Solomon AW, Engels D, Bailey RL, Blake IM, Brooker S, Chen JX,et al. A diagnostics platform for the integrated mapping, monitoring, and surveillance of neglected tropical diseases: rationale and target product profiles. PLoS Negl Trop Dis 2012;6:e1746.
66. Steinmann P, Keiser J, Bos R, Tanner M, Utzinger J. Schistosomiasis and water resources development: systematic review, meta-analysis, and estimates of people at risk. Lancet Infect Dis. (2006) 6:411–25. doi: 10.1016/S1473-3099(06)70521-7
67. Tchuem Tchuenté LA, Kuete Fouodo CJ, Kamwa Ngassam RI, Dongmo Noumedem C, Kenfack CM, Gipwe NF, et al. Evaluation of circulating cathodic antigen (CCA) urine-tests for diagnosis of Schistosoma mansoni infection in Cameroon. PLoS Negl Trop Dis 2012;6: e1758.
68. The Red Urine Study Group. Identification of high-risk communities for schistosomiasis in Africa: a multicountry study. SER Project Reports. Geneva: World Health Organization; 1995. p. 1–103.
69. Tidi, S.K.; Jummai, A.T. Urinary schistosomiasis: Health seeking behaviour among residents of Kiri in Shelleng Local Government Area of Adamawa state. J. Environ. Toxicol. Publ. Health 2015, 1, 30–35.
70. Uchendu O, Oladoyin V, Idowu M, Adeyera O, Olabisi O, Oluwatosin O, et al. Urinary schistosomiasis among vulnerable children in a rehabilitation home in Ibadan, Oyo state, Nigeria. BMC Infect Dis. (2017) 17:487. doi: 10.1186/s12879-017-2591-6
71. Umeh JC, Amali O, Umeh EU. The socio-economic effects of tropical diseases in Nigeria. Econ Hum Biol. (2004) 2:245–63. doi: 10.1016/j.ehb.2004.04.001
72. Uneke CJ, Ezeoha AE, Uro-Chukwu H, Ezeonu CT, Ogbu O, Onwe F, et al. Enhancing the capacity of policy-makers to develop evidence-informed policy brief on infectious diseases of poverty in Nigeria. Int J Health Policy Manag. (2015) 4:599–610. doi: 10.15171/ijhpm.2015.100
73. Utzinger J, Becker SL, van Lieshout L, van Dam GJ, Knopp S. New diagnostic tools in schistosomiasis. Clin Microbiol Infect. 2015 Jun;21(6):529-42. doi: 10.1016/j.cmi.2015.03.014. Epub 2015 Apr 3. PMID: 25843503.
74. Valenta AL and Wigger U. Q-methodology: definition and application in health care informatics. Journal of the American Medical Informatics Association 1997;4(6):501–10. Available from: <https://doi.org/10.1136/jamia.1997.0040501>.
75. van Dam GJ, de Dood CJ, Lewis M, Deelder AM, van Lieshout L, Tanke HJ, et al. A robust dry reagent lateral flow assay for diagnosis of active schistosomiasis by detection of Schistosoma circulating anodic antigen. Exp Parasitol 2013;135:274 – 82.
76. Van G-Y, Onasanya A, van Engelen J, Oladepo O, Diehl JC. Improving access to diagnostics for schistosomiasis case management in Oyo State, Nigeria: barriers and opportunities. Diagnostics. (2020) 10:328.doi: 10.3390/diagnostics10050328
77. van Lieshout L, Roestenberg M. Clinical consequences of new diagnostic tools for intestinal parasites. Clin Microbiol Infect 2015 [in this issue].
78. Van Woezik AFG, Braakman-Jansen LMA, Kulyk O, Siemons L, van Gemert-Pijnen JEWC. Tackling wicked problems in infection prevention and control: a guideline for co-creation with stakeholders. Antimicrob Resist Infect Control. (2016) 5:20. doi: 10.1186/s13756-016-0119-2
79. Verweij JJ, Stensvold CR. Molecular testing for clinical diagnosis and epidemiological investigations of intestinal parasitic infections. Clin Microbiol Rev 2014;27:371 – 418.
80. Weber MD, Blair DM, Clark VV. The pattern of schistosome egg distribution in a micturition flow. Cent Afr J Med. (1967) 13:75–88.
81. WHO. Preventive chemotherapy in human helminthiasis: coordinated use of anthelmintic drugs in control interventions: a manual for health professionals and programme managers. Geneva: World Health Organization; 2006. p. 1–74.
82. [WHO. Schistosomiasis: progress report 2001–2011 and strategic plan 2012–2020. Geneva: World Health Organization; 2013. p.1–80.](https://docs.google.com/document/d/1OuODgPGH3bLqyIAdcXAlcPH1jYGb_q3m4gTNmaTFb4E/edit#bookmark=id.ddvn8yw700uj)
83. World Health Organization. A Roadmap for Implementation: Accelerating Work to Overcome the Global Impact of Neglected Tropical Diseases. Geneva: World Health Organization (2012).
84. World Health Organization. Malaria Rapid Diagnosis — Making it Work. Informal consultation on field trials and quality assurance of malaria rapid diagnostic tests. WHO meeting report, 20–23 Jan 2003. [<[http://www.wpro.who.-](about:blank) int/rdt/docs/RDTs–MakingItWork.pdf>].
85. World Health Organization. Schistosomiasis: number of people treated worldwide in 2018. Wkly Epidemiol RecWER. (2019) 94:601–12. Available online at: <https://www.who.int/wer/2019/wer9450/en/>
86. World Health Organization. Schistosomiasis Fact Sheet. World Health Organization (2020). Available online at: <https://www.who.int/news-room/> fact- sheets/detail/schistosomiasis (accessed October 5, 2020).
87. Worrell CM, Bartoces M, Karanja DMS, Ochola EA, Matete DO, Mwinzi PNM, et al. Cost analysis of tests for the detection of Schistosoma mansoni infection in children in western Kenya. AmJ TropMed Hyg. (2015) 92:1233–9. doi: 10.4269/ajtmh.14-0644
88. Solomon AW, Engels D, Bailey RL, et al. A diagnostics platform for the integrated mapping, monitoring, and surveillance of neglected tropical diseases: rationale and target product profiles. PLoS Negl Trop Dis. 2012;6(7):e1746
89. Bergquist R, Johansen MV, Utzinger J. Diagnostic dilemmas in helminthology: what tools to use and when? Trends Parasitol. 2009;25(4):151–156.
90. World Health Organization. Helminth control in school-age children: A guide for managers of control programmes, Second Edition. 2011.
91. Uniting to Combat Neglected Tropical Diseases. Delivering on promises and driving Progress. 2014. Available at: unitingtocombatntds.org/resource/delivering-promises-and-driving-progress-second-progress-report.
92. PATH. Diagnostics for neglected tropical diseases: Defining the best tools through target product profiles. Seattle: PATH; 2015.
93. PATH. Target Product Profile. Schistosomiasis Surveillance Diagnostic. Use case: Preventive chemotherapy reductionor stopping decision. General to all platforms and biomarkers. Seattle: PATH; 2015.
